# Earliest Jurassic plant assemblages from Sweden reveal a low-diversity ginkgoalean and cheirolepid flora dominating the post-extinction landscape

**DOI:** 10.1093/aob/mcaf143

**Published:** 2025-07-09

**Authors:** Daniela Quiroz-Cabascango, Vivi Vajda, Stephen McLoughlin, Grzegorz Niedźwiedzki

**Affiliations:** Department of Palaeobiology, Swedish Museum of Natural History, SE-114 18 Stockholm, Sweden; Department of Ecology, Environment and Plant Sciences, Stockholm University, SE-104 05 Stockholm, Sweden; Department of Palaeobiology, Swedish Museum of Natural History, SE-114 18 Stockholm, Sweden; Department of Palaeobiology, Swedish Museum of Natural History, SE-114 18 Stockholm, Sweden; Department of Organismal Biology, Uppsala University, SE-752 36 Uppsala, Sweden; Polish Geological Institute-National Research Institute, 00-975 Warszawa, Poland

**Keywords:** Palaeoecology, Boserup beds, North Atlantic floral province, mass extinction, Ginkgoopsida, Cheirolepidiaceae, Osmundaceae, Equisetales

## Abstract

**Background and Aims:**

Rich Triassic–Jurassic plant assemblages from Skåne, southern Sweden, have been documented extensively over the past two centuries. However, no macrofloras from the lowermost part of the Helsingborg Member (Lower Jurassic) have been forthcoming and thus the age of the successions has not been well constrained. Here we systematically describe and assess the palaeoecology and age of a newly discovered flora from the Boserup beds at Norra Albert Quarry, Skåne.

**Methods:**

Plant macrofossils were examined using macrophotography, fluorescence microscopy and scanning electron microscopy. Palynological analysis of the strata hosting the macroflora contributed to the palaeoenvironmental interpretations and refined the age of the deposits.

**Results:**

The low-diversity post-extinction recovery forests of the earliest Jurassic were dominated by ginkgoopsids, cheirolepid conifers and ferns, growing under seasonal mesothermal conditions. Dispersed charcoal indicates wildfires were present in the landscape at this time.

**Conclusions:**

Despite the poor preservation of the fossils, the Boserup beds flora provides a window into vegetation recovery in the immediate aftermath of the end-Triassic extinction event. Initial recovery is characterized by the presence of needle- and scale-leafed seed plants (notably czekanowskialeans and *Brachyphyllum* species that produced *Classopollis* pollen), along with a range of ground ferns.

## INTRODUCTION

The end-Triassic extinction event (ETE), 201 million years ago (Ma), is one of the ‘big five’ biotic crises of the Phanerozoic (Sepkoski, 1981; McElwain *et al.*, 2007; Wignall and Atkinson, 2020). The primary driver of this event is typically attributed to extensive flood basalt volcanism in the Central Atlantic Magmatic Province (CAMP), which released large quantities of greenhouse gases into the atmosphere, triggering ocean acidification and significant changes in global temperatures, either abrupt warming or cooling, depending on the data and palaeoclimate modelling approach employed (Marzoli *et al.*, 1999; McElwain *et al.*, 1999; van de Schootbrugge and Wignall, 2016; Capriolo *et al.*, 2020; Wignall and Atkinson, 2020; Landwehrs *et al.*, 2021; Schoepfer *et al.*, 2022; Olsen *et al*., 2022).

These environmental perturbations caused a significant ecological turnover affecting both marine and terrestrial communities. Marine organisms, such as bivalves, brachiopods, ostracods, calcareous algae and radiolarians, were heavily affected (Wignall and Atkinson, 2020). On land, notably the seed-plant family Peltaspermaceae became globally extinct (McElwain *et al.*, 2007; Mander *et al.*, 2010; Kustatscher and van Konijnenburg-van Cittert, 2013; Slodownik *et al.*, 2021), apart from a few relictual representatives that survived at high southern palaeolatitudes (Patagonia and Australia) into the Early Jurassic (Bomfleur *et al*., 2018; Elgorriaga *et al.*, 2019). The response of the global terrestrial vegetation to the environmental perturbations during the ETE has been broadly studied and discussed, and these responses appear to have varied geographically (McElwain *et al.*, 1999, 2007; McElwain and Punyasena, 2007; van de Schootbrugge *et al.*, 2009; Mander *et al.*, 2010; Barbacka *et al.*, 2014*a*, 2017; Li *et al.*, 2016; Bos *et al.*, 2023; Vajda *et al*., 2023). Therefore, analysis of the Early Jurassic floras from multiple regions is essential for understanding how plant communities of various biomes recovered under conditions of environmental stress.

Several localities in the northern European–North Atlantic region offer prime opportunities to study Early Jurassic fossil floras from the aftermath of the ETE. These include the Early Jurassic floras of the Holy Cross Mountains, Poland (Pacyna, 2013; Barbacka *et al*., 2014*b*), the Mecsek Mountains, southern Hungary (Barbacka, 2011), Franconia, Germany (Weber, 1968), and Scoresby Sound, East Greenland (Harris, 1931, 1935; Lundblad, 1950; McElwain *et al*., 2007). The rich Late Triassic–Early Jurassic (Rhaetian–Sinemurian) macrofloral assemblages of Skåne (southern Sweden) are known to incorporate diverse ‘pteridosperms’, ginkgoopsids, conifers, bennettitaleans, ferns, and other less abundant plant groups (Nathorst, 1878; Antevs, 1919; Troedsson, 1947; Lundblad, 1950, 1959*a*; Guy-Ohlson and Norling, 1994; Pott and McLoughlin, 2009, 2011; Krüger *et al*., 2021; Vajda *et al*., 2023). However, the macrofloras in the lowermost part of the Höganäs Formation (the Boserup beds) have been overlooked owing to the sparse occurrence and poor preservation of plant remains within thin siltstone laminae set in coarse-grained sandstones. These ambiguously dated beds have commonly been denoted as the ‘Transitional interval’ spanning the Triassic/Jurassic boundary (Larsson, 2009; Lindström *et al*., 2017; Krüger *et al*., 2021; Vajda *et al.*, 2023).

Here, we systematically describe a newly discovered plant assemblage recovered from the lowermost part of the Boserup beds that fills the stratigraphic gap between the rich Rhaetian (Upper Triassic) and upper Hettangian–Sinemurian (Lower Jurassic) plant suites of the region. The macroflora supported by palynological analyses of the macrofossil-bearing strata provide precise biostratigraphic placement of the beds. The palynoassemblages also help link dispersed spores/pollen to their respective parent plants. As this flora is no longer accessible owing to recent cessation of pumping operations and inundation of the quarry by groundwaters, these collections become increasingly valuable, significantly contributing to the broader understanding of the regional vegetation that recovered immediately after the ETE. Furthermore, the Boserup beds flora offers important insights into the age of the successions and the emergence and adaptation of plant communities under the stressed climatic conditions of the earliest Jurassic.

## MATERIALS AND METHODS

**Fig. 1.**
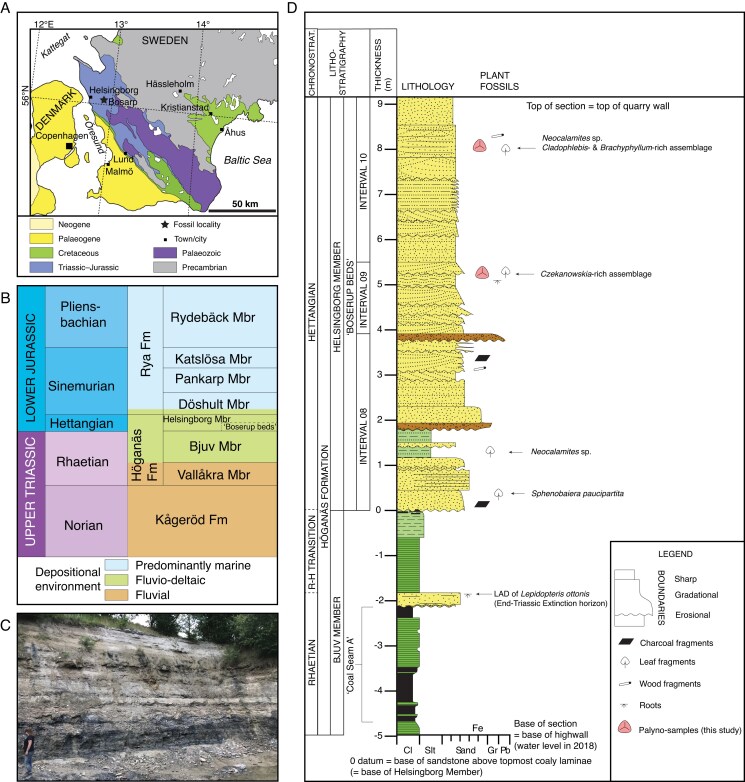
Geological setting of the study area. (A) Geological map of Skåne, southern Sweden, indicating the location of the Boserup beds exposure at Norra Albert Quarry. (B) Stratigraphy of the uppermost Triassic (Rhaetian) and Lower Jurassic succession in southern Sweden. Modified from Petersen *et al*. (2013). (C) Norra Albert outcrop showing the coal-bearing Bjuv Member (base of section) and Boserup beds (sandy top of section). (D) Stratigraphic column of Norra Albert Quarry showing sampling levels. Modified from Vajda *et al*. (2023). Fm, formation; LAD, last appearance datum; Mbr, member.

The Höganäs Formation (Upper Triassic–Lower Jurassic) is distributed in several sub-basins in northwestern and southwestern Skåne, along the margin of the Danish Basin (Fig. 1A). It comprises the Vallkra and Bjuv members, dated as Rhaetian (∼208.5 or 205.7–201.4 Ma), and the Helsingborg Member dated as Hettangian (∼201.4–199.5 Ma) (Fig. 1B; Ahlberg *et al.*, 2003; Lindström and Erlström, 2006; Larsson, 2009; Vajda *et al.*, 2023). The Helsingborg Member comprises mainly carbonaceous mudstones, siltstones, sandstones and heterolithic beds deposited in marginal marine and non-marine settings (Pieńkowski, 1991; Vajda and Wigforss-Lange, 2009; Peterffy *et al*., 2016). Here we focus on the succession at the base of the Helsingborg Member, comprising very immature arkoses separated by pale claystones with sideritic bands and nodules (Sivhed, 1984; Pieńkowski, 1991) widespread across northwestern Skåne (Troedsson, 1951). These beds were informally designated the ‘Boserup beds’ (lowermost Hettangian) within the Helsingborg Member (Fig. 1C, D) by Troedsson (1951) and have been interpreted as deposits of meandering (Pieńkowski, 1991) or braided (Weibel *et al*., 2016) rivers.

Fossiliferous intervals in the lower part of the Boserup beds sampled in this study are represented by silty, medium to dark grey or olive carbonaceous mudstones with abundant plant fragments, generally occurring as imprints. Although the age of this unit has been considered ambiguous, the overlying parts of the Helsingborg Member are considered to represent Hettangian (Lower Jurassic) deposits (Pieńkowski, 1991; Ahlberg *et al*., 2003).

Macrofossil and palynological samples were collected from the sedimentary successions exposed on the western and southern wall of the Norra Albert Quarry, informally referred to in some studies as ‘Schakt Albert’ or S. Vram. Unfortunately, this sampled section is no longer accessible as quarrying and consequently the pumping of groundwater ceased in 2022 leading to inundation of the quarry (Fig. 1C). This study focuses on plant fossils recovered from four fossiliferous beds located in the basal 10 m of the Boserup beds within the Helsingborg Member (Fig. 1D).

### Macroflora

The plant macrofossils were identified from three mudstone layers, intervals 8 (0–4 m), 9 (4–5.5 m) and 10 (5.5–9 m) (Fig. 1D), and are stored at the Museum of Evolution of Uppsala University (PMU). All specimens were collected by G.N. between 2018 and 2020 (PMU 35641, PMU 34512, PMU 34477, PMU 34515, PMU 34509, PMU 32551, PMU 32594, PMU 32552, PMU 34542, PMU 32588, PMU 34541, PMU 35642, PMU 35643, PMU 32572, PMU 32591, PMU 32568, PMU 32560, PMU 32544, PMU 32547, PMU 32600, PMU 34549, PMU 34532, PMU 34454, PMU 34525, PMU 34496, PMU 34540, PMU 34110, PMU 35644, PMU 32561). Each specimen was studied, identified and photographed under strong unilateral light using Canon EOS 2000D and Canon EOS 5DSR cameras, an Olympus SZX10 stereomicroscope equipped with an Olympus DP-71 digital camera or, where higher-resolution images were required of cuticular details, with Olympus BX51 and Leica DM 2000 transmitted light microscopes equipped with Lumenera Infinity 2 and Leica DFC310 FX digital cameras, respectively. Microscopic features were measured using cellSens^©^ Dimension version 1.6 (Olympus Soft Imaging Systems; Münster, Germany). For improved clarity of anatomical features, stacking of images from multiple focal planes was undertaken using Adobe Photoshop CC 2019 software.

A few macrofossils retaining cuticle were studied using fluorescence microscopy (Olympus BX51 microscope with incident UV light excitation at ∼460–490 nm) because UV light has the potential to highlight epidermal cells and stomatal patterning through autofluorescence even on degraded compressions.

An FEI Quanta FEG 650 environmental scanning electron microscope at the Swedish Museum of Natural History was utilized to obtain fine-scale morphological features of selected plants.

### Palynological processing and analysis

Two sedimentary samples were collected for palynological analysis in order to link elements of the flora to the pollen they produced, resolve the age of the macroflora-bearing lower part of the Boserup beds, clarify the depositional environment, and provide broader insights into the composition and ecology of the palaeovegetation.

One sample was collected from interval 9 hosting *Czekanowskia* leaves (PMU 32554), and another was obtained from interval 10 hosting *Brachyphyllum* remains (PMU 34496). These samples were processed at Global GeoLab Ltd (Medicine Hat, Alberta, Canada) using standard palynomorph extraction methods (for detailed description see Vajda and Kear, 2024). Treatment started by placing 12 g of sample in a 400-mL polypropylene beaker followed by adding 10 % solution of HCl overnight allowing time for carbonates to dissolve. Next, 70 % HF was added and the sample oscillated for up to 4 h until dissolution was completed. The residue was poured into a 50-mL polypropylene test tube and the following steps involved heavy liquid separation of organic remains using ZnBr_2_, oxidation by Schultze solution and, finally, sieving the washed residue through a 5-μm nylon mesh. Slides were prepared by mixing the sieved residue with one drop of polyvinyl alcohol and clear casting; a coverslip was applied and the mount was sealed. Two slides were examined for presence/absence data, and 100 palynomorphs counted to establish relative abundances (Table 1). The palynofacies analyses involved relative abundance counts of 500 organic particles categorized as phytoclasts (mainly wood particles), cuticle fragments, pollen, spores and algae (Table 2). All palynological and kerogen samples examined for this study are publicly accessible through the PMU collections (slides PMU 32554-01 and PMU 34496-01).

**Table 1.** Palynomorph taxa identified in the ‘*Brachyphyllum* bed’ interval 10 from Boserup beds.

Palynomorph taxa	%
Bryophytes	
*Stereisporites psilatus*	1
Lycophyte	
*Aratrisporites minor*	7
*Calamospora mesozoica*	2
Ferns	
*Cyathidites minor*	9
*Deltoidospora toralis*	8
*Osmundacidites wellmanii*	2
*Todisporites* sp.	4
Gymnosperms	
*Alisporites thomasii*	2
*Classopollis minor*	55
*Chasmatosporites apertus*	2
*Monosulcites* sp.	2
*Pinuspollenites minimus*	3
Algae	3
Total	100

**Table 2.** Categories of organic matter present in the sample from the *Czekanowskia* bed interval 9 and in the sample from the ‘*Brachyphyllum* bed’ interval 10. Percentages are based on counts of 500 particles per sample.

Palynofacies types (%)	Interval 9	Interval 10
Opaque phytoclasts	94	4
Translucent phytoclasts	0	84
Cuticles	6	11
Pollen	0	1
Spores	0	0
Total organic matter	100	100

## RESULTS

Plant fossils from the Boserup beds are preserved mainly as poor-quality impressions and coalified compressions. The fossil flora consists of leaf fragments, seeds, roots and pollen-bearing structures. In this study, only *Brachyphyllum* and *Sphenobaiera* yielded taxonomically informative features. The generally poor state of preservation hampers identification to species level for most of the leaf-based taxa, as crucial diagnostic features, such as venation patterns and the proximal and distal portions of the laminae, are commonly obscured or absent (Fig. 9F, G). As a result, the majority of the specimens described here have been assigned to well-established fossil genera but few can be identified unequivocally to species.

### Systematic palaeobotany

Class. Polypodiopsida

Order. Equisetales

Family. *Incertae sedis*

Genus. ***Neocalamites***Halle, 1908.

*Type species*. *Neocalamites lehmannianus* (Göppert, 1846) Weber, 1968; Rhaetian, Silesia.

**Fig. 2.**
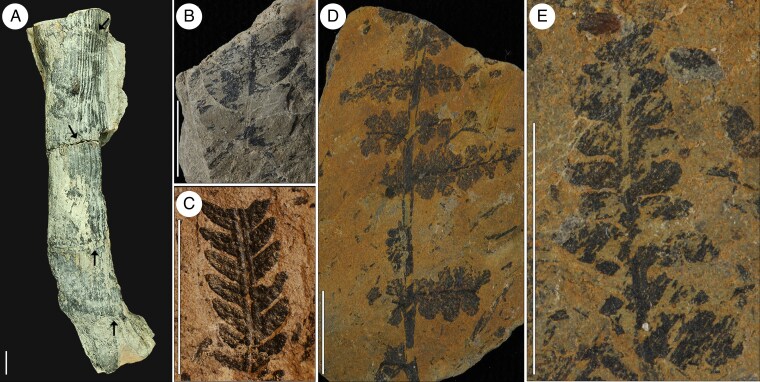
(A) *Neocalamites* sp. axis with transverse nodes (arrows); PMU 35641. (B) *Cladophlebis* spp. Pinna fragment having falcate pinnules with acute apices and decurrent bases; PMU 34512. (C) *Cladophlebis* spp. pinna with pronounced midvein; PMU 34477. (D) *Cladophlebis* spp. frond fragment with sub-opposite pinnae; PMU 34515. (E) *Cladophlebis* spp. pinna fragment, bearing pinnules with rounded apices; PMU 34509. Scale bar = 10 mm.

*Neocalamites* sp. (Fig. 2A)

*Material*. Four specimens: PMU 35641 (interval 8); PMU 34501, PMU 34480, PMU 34532(a) (interval 10), Boserup beds.

*Description*. Near-cylindrical, longitudinally ribbed, jointed stem cast (Fig. 2A), 150 mm long, 40 mm wide. The apex, base and leaves are not preserved. Cast surface bearing 17–20 ribs, each 2–3 mm wide. Four swollen nodes visible on the studied axes (arrows in Fig. 2A). Internodes 35–45 mm long.

*Remarks*. *Neocalamites* ranges from the uppermost Permian to Lower Cretaceous (Harris, 1961; Pott and McLoughlin, 2009), although essentially identical articulated axes from the Cretaceous and Cenozoic are typically assigned to *Equisetites* Sternberg (Kva”ek and Čerňanský, 2024) or *Equisetum* L. (Pole and McLoughlin, 2017; Rozefelds *et al*., 2019). On this basis, such axes have little stratigraphic utility. The Norra Albert specimens are similar to *Neocalamites* sp. axes reported by Mehlqvist *et al*. (2009) from the Early Jurassic (Pliensbachian) flora of Bornholm, Denmark. The genus is well represented in Rhaetian–Lower Jurassic assemblages of Skåne (Halle, 1908; Antevs, 1919; Lundblad, 1950) and some axes have been found associated with shark eggs in the Helsingborg Member, indicating that the partly submerged stems of these plants may have provided important nurseries for Early Jurassic aquatic vertebrates (Krüger *et al*., 2021).

Order. Osmundales

Family. Osmundaceae

Genus. ***Cladophlebis***Brongniart, 1849.

*Type species*. *Cladophlebis albertsii* (Dunker) Brongniart, 1849; Early Cretaceous, northern Germany.

*Cladophlebis* spp. (Fig. 2B–E)

*Material*. Five specimens: PMU 34512 (interval 9); PMU 34477, PMU 34509, PMU 34515 (interval 10), Boserup beds.

*Description*. Preserved portions of fronds 46 mm long and 22 mm wide (Fig. 2D) consisting of opposite to sub-opposite pinnae attached to a 1.2-mm-wide central rachis. Pinnae bearing alternate (Fig. 2B) or sub-opposite (Fig. 2E), lanceolate, short pinnules (5–20 mm long and 5–10 mm wide) with entire margins (Fig. 2C) and decurrent bases. Most apices rounded (Fig. 2E) or distally inclined/arched (Fig. 2B). Midvein of larger pinnules well defined (0.9 mm thick; Fig. 2C), deriving from rachilla and persisting to pinnule apex. Secondary veins indistinct. Midvein of shorter pinnules ill-defined (e.g. PMU 34515) and secondary veins radiate and bifurcate from near pinnule base (Fig. 2D).

*Remarks*. *Cladophlebis* is widely employed for Mesozoic fern foliage and was defined by Brongniart (1849) to accommodate only dissected sterile fronds (Monje-Dussán *et al*., 2016). It is characterized by linear or falcate ultimate segments attached to the rachilla by the whole of their base; possessing a midvein that is prominent basally but evanescing distally into finer secondary veins at an acute angle (Seward, 1910). Fertile fronds, morphologically equivalent to *Cladophlebis*, are traditionally assigned to *Todites* Seward but no obvious sori were evident on the Norra Albert specimens.

Two morphotypes of *Cladophlebis* pinnae are apparent in the assemblage: one with inclined falcate pinnules having prominent midribs (Fig. 2B, C), and another with oblong pinnules inserted at nearly 90° to the rachilla. However, given the fragmentary nature of the specimens and the variability in pinna form within extant Osmundaceae fronds, it is not possible to resolve whether these morphotypes represent different species or constitute intraspecific variants in pinna form.

Although the shape and arrangement of the pinnules in the Norra Albert specimens are consistent with *Cladophlebis svedbergii*Johansson (1922), described from uppermost Triassic–lowermost Jurassic strata at Stabbarp and Skromberga, southern Sweden, confident attribution to that species, or others in the genus, is not possible owing to poor preservation of the venation in the Norra Albert material.

Class. Ginkgoopsida (*sensu*Anderson *et al*., 2007)

Order. Czekanowskiales Novák, 1972 (= Leptostrobales Meyen, 1984)

Genus. ***Czekanowskia*** Heer, 1876

*Type species*. *Czekanowskia setacea*, Heer, 1876; Middle Jurassic, eastern Siberia.

**Fig. 3.**
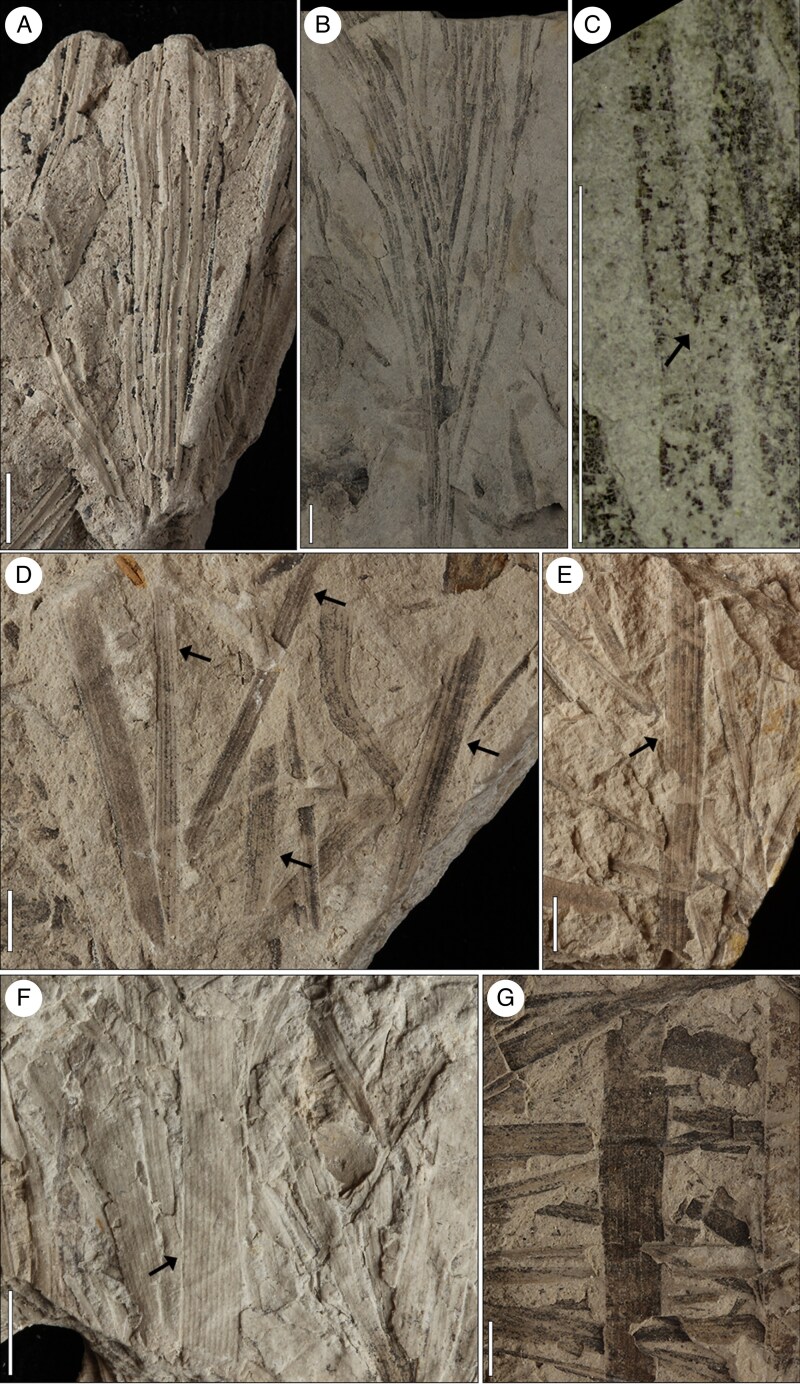
Czekanowskialean and ginkgoalean leaf fragments. (A) *Czekanowskia* sp. leaf cluster impression preserving minimal carbonized organic remnants; PMU 32551. (B) *Czekanowskia* sp. leaf cluster; PMU 32594. (C) Simple forked *Czekanowskia* sp. leaf; PMU 32594. (D) *Pseudotorellia* sp. leaves (arrows); PMU 32552. (E) Strap-shaped *Pseudotorellia* sp. leaf with parallel venation; PMU 34542. (F) *Ginkgoites* sp. cf. *G*. *marginatus* linear leaf fragment bearing 10 parallel veins; PMU 32588. (G) *Ginkgoites* sp. cf. *G*. *marginatus* linear leaf segment; PMU 34541(a). Scale bar = 5 mm.

*Czekanowskia* sp. (Fig. 3A–C)

*Material*. Two specimens: PMU 32551, PMU 32594 (interval 9), Boserup beds.

*Description*. Leaves forked into two equal filiform, entire-margined segments (Fig. 3C), 40–50 mm long, 1.4 mm wide, each with a midvein. Leaves arranged in a tight cluster of 11–16 inserted on a short shoot (Fig. 3A, B).

*Remarks*. *Solenites* species differ from *Czekanowskia* sp. from the Boserup beds in having unforked leaves (Harris and Miller, 1974). The Boserup leaves share morphological similarities, such as linear lamina segments, with some species of *Baiera* (Bauer *et al*., 2013), but the latter are more fan-shaped and possess a distinct petiole, which is lacking in *Czekanowskia*.

Similar fossils to those attributed here to *Czekanowskia* sp. were described as *Czekanowskia hartzii* T.M. Harris and *C. nathorstii* T.M. Harris from basal Jurassic strata in East Greenland (Harris, 1935). Those leaves, all sourced from the same bed, are clearly organized in bundles of approximately eight. Leaves are typically around 200 mm long and 0.4–1.4 mm wide and, given their overlap in macromorphological and epidermal characters, may represent a single species. Both Greenland forms have acute apices and thick cuticles that yielded details of epidermal patterning (Harris, 1935). The studied specimens are difficult to differentiate from either of the Greenland forms in the absence of these characters.

Order. Ginkgoales Gorozhankin, 1904

Family. Pseudotorelliaceae Krassilov, 1972

Genus. ***Pseudotorellia*** Florin, 1936

*Type species*. *Pseudotorellia nordenskioeldii* (Nathorst) Florin, 1936; Upper Jurassic, Spitsbergen.

*Pseudotorellia* sp. (Fig. 3D, E)

*Material*. Two specimens: PMU 32552, PMU 34542 (interval 9), Boserup beds.

*Description*. Linear leaf fragments, entire-margined, containing four or five parallel veins (arrows in Fig. 3D, E). Complete length unknown, fragments up to 90 mm long and 3–5 mm wide. The apices and bases of leaves are not preserved; margin entire.

*Remarks*. Although specimens of this taxon are relatively common in some beds, none occur in arrangements that suggest divergence from a common petiole, as in *Baiera*, *Sphenobaiera* or *Ginkgoites*. Instead, they consistently occur as relatively large, isolated, parallel-margined laminae, which suggests they represent entire linear leaves.

The cuticles of the Norra Albert specimens are poorly preserved and no complete leaf was found. Therefore, the leaf size and shape and number of parallel veins favours assignment to *Pseudotorellia* but there are insufficient details to support assignment to a formal species.

Family. Ginkgoaceae Engler (in Engler and Prantl, 1897)

Genus. ***Ginkgoites*** Seward, 1919

*Type species*. *Ginkgoites sibirica* (Heer) Seward, 1919; Middle Jurassic, East Siberia.

***Ginkgoites* sp. cf. *G*. *marginatus*** (Nathorst) Florin (Fig. 3F, G)

*Material*. Two specimens: PMU 32588, PMU 34541(a) (interval 9), Boserup beds.

*Description*. Linear, entire-margined leaf fragments (Fig. 3F), 20–50 mm long, 4–5 mm wide, containing 7–10 parallel, sparsely dichotomizing veins (Fig. 3G); apex and base not preserved; margin entire.

*Remarks*. Determining the identification of these lamina fragments is challenging based on the few available morphological features and, in the absence of epidermal characters. Several genera, such as *Pseudotorellia*, *Podozamites*, *Phoenicopsis*, *Desmiophyllum* and *Ginkgoites*, embrace features of these fossils. However, *Pseudotorellia* can be excluded by its lesser vein density. The emended diagnosis of *Podozamites* proposed by Shi *et al*. (2017) encompasses ‘leaves narrowly oblong to strap shaped, more or less symmetrical, with entire margins and crowded parallel veins’. Similarly, *Desmiophyllum* is characterized by simple, elongate leaves (150 mm long and 25 mm wide) with 25–36 parallel veins per leaf (Schweitzer, 1977). Although the fragments from the Boserup beds are linear, they differ in having markedly fewer (7–10) veins than *Podozamites*, *Desmiophyllum* or most *Phoenicopsis* species.

The linear leaves of *Phoenicopsis* are typically arranged in clusters of six attached to a short shoot and feature 10–12 parallel veins per 5 mm with one to three bifurcations at the proximal end (Sun *et al.*, 2015). Generally, *Phoenicopsis* leaves are larger and with greater vein densities than the specimens illustrated herein.

Lundblad (1959*b*) described the holotype of *Ginkgoites marginatus* from the Lower Jurassic Helsingborg Member at Helsingborg, Skåne (southern Sweden) as having fan-shaped leaves dissected into eight linear segments. The margins of each segment are parallel in the central portion but converge basally and apically. The leaves documented by Lundblad (1959*b*) have maximum widths of ∼5.5 mm and contain five or six veins, corresponding to a vein separation of ∼0.8 mm, and a vein density of ∼12 per centimetre. Based on these similarities, the specimens from the Boserup beds probably represent fragmented segments of *Ginkgoites marginatus* leaves, and this is consistent with the geographic and stratigraphic proximity of the Norra Albert and Helsingborg deposits.

Order. Ginkgoales

Genus. ***Sphenobaiera*** Florin, 1936, emend. Harris and Millington, 1974

*Type species*. *Sphenobaiera spectabilis* (Nathorst) Florin, 1936; Jurassic, Franz Josef Land.

**Fig. 4.**
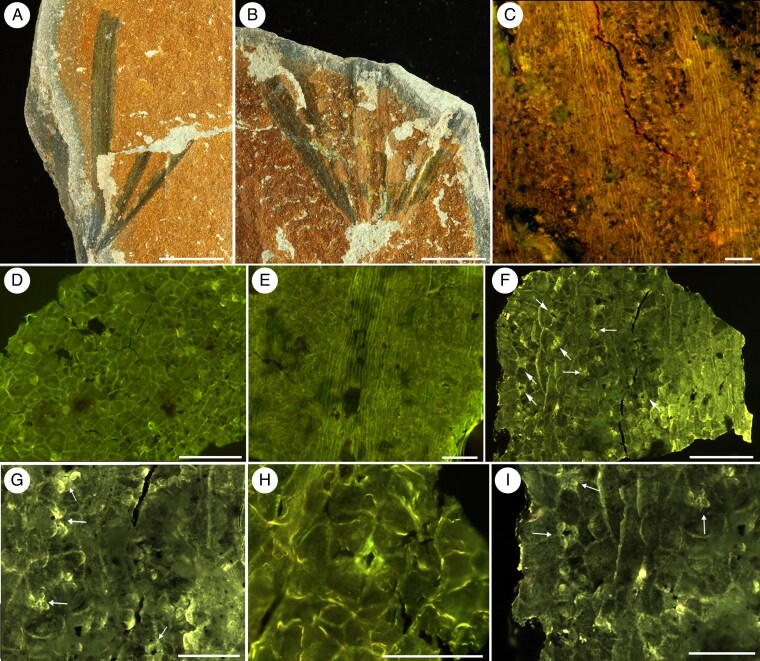
*Sphenobaiera paucipartita* leaves. (A) Leaf with three linear segments preserved; PMU 35642. (B) Deeply dissected leaf bearing at least five segments; PMU 35643. (C) Abaxial cuticle bearing well-defined stomata and non-stomata zones; PMU 35643. (D) Adaxial cuticle showing isodiametric epidermal cells; PMU 35643. (E) Ten to twelve longitudinal elongated cells over veins; PMU 35643. (F) Abaxial cuticle with papillae overhanging stomatal complex (arrows); PMU 35643. (G) Outer view of a stoma showing thickened subsidiary cells and papillae (arrows) overhanging the stomata pit; PMU 35643. (H) Inner surface of abaxial cuticle bearing weakly developed papillate overhanging stoma; PMU 35643. (I) Papillae (arrows) overhanging the stomatal pit; PMU 35643. Scale bar (A–C) = 1 cm; (D–F) = 100 μm; (G–I) = 50 μm.

***Sphenobaiera paucipartita*** (Nathorst) Florin (Fig. 4A–I)

*Material*. Two specimens. PMU 35642, PMU 35643 (interval 8), Boserup beds.

*Description*. Fan-shaped, deeply dissected leaf with at least five segments narrowing towards the base (Fig. 4B); each segment with four parallel veins and entire margins. The original size is indeterminate owing to the absence of the apex, base and petiole of the leaves (Fig. 4A, B). Preserved portions of lobes are 35 mm long and 2–3 mm wide.

Leaves hypostomatic. Adaxial surface with isodiametric, rectangular and polygonal epidermal cells (Fig. 4D). Stomatal and non-stomatal zones well defined in the abaxial cuticle (Fig. 4C). Non-stomatal zones are characterized by veins composed of 8–12 rows of longitudinally elongated, rectangular epidermal cells (Fig. 4E). In contrast, the stomatal zones consist of isodiametric, polygonal to short rectangular cells, with well-defined straight cell walls (Fig. 4F–I). Stomata distributed between the vein courses, forming poorly defined longitudinal bands (Fig. 4F). Stomatal complexes incorporating four to six subsidiary cells bearing papillae that project towards the stomatal aperture, obscuring the sunken guard cells (Fig. 4G–I).

*Remarks*. The new material is broadly consistent with the characters of *Sphenobaiera*, *Ginkgoites* and *Baiera*. The most important features differentiating these taxa are the shape of the leaf base (presence/absence of petiole) and the number of veins at the leaf base, one vein in *Sphenobaiera* and two in *Ginkgoites* (Taylor *et al.*, 2009; Barbacka, *et al.*, 2014*b*). These features are difficult to observe in the present material owing to the poor state of preservation. However, the general morphology of the leaves indicates affiliation with *Sphenobaiera*, which is characterized by wedge-shaped, deeply dissected leaves with dichotomous venation and the absence of a distinct petiole (Harris and Millington, 1974; Bauer *et al.*, 2013).

The Boserup beds specimens are similar to *Sphenobaiera leptophylla* Harris from the Rhaetian of East Greenland and *Sphenobaiera insecta* J.M. Anderson et H.M. Anderson from the Upper Triassic of Australia (Harris, 1935; Unverfärth *et al*., 2022). However, the Greenland and Australian forms differ in lacking resin bodies and having amphistomatic cuticles (Lundblad, 1959*b*; Harris and Millington, 1974; Unverfärth *et al.*, 2022)

*Sphenobaiera spectabilis*, reported from Hettangian strata at Stabbarp (Sweden), Scoresby Sound (East Greenland) and the Holy Cross Mountains (Poland) can be distinguished by its amphistomatic cuticle with approximately equal stomatal densities on both leaf surfaces (Lundblad, 1959*b*; Pacyna, 2013; van Konijnenburg-van Cittert *et al.*, 2022). Likewise, *Sphenobaiera huangii* (Sze) Hsü from the Lower Jurassic of Hubei, China, differs from the Boserup material in possessing an amphistomatic cuticle with five to eight subsidiary cells per stoma (Wang *et al*., 2005*a*).

This material best matches *Sphenobaiera paucipartita* from the Rhaetian of Skåne, Sweden, and *Sphenobaiera boeggildiana* Harris from Rhaetian strata of Jameson Land, East Greenland and Bavaria, Germany (Harris, 1935; Lundblad, 1959*b*; van Konijnenburg-van Cittert *et al.*, 2022). Both species are imperfectly hypostomatic with well-defined stomatiferous zones, and stomata surrounded by subsidiary cells bearing papillae that project towards the stomatal aperture. However, *S. boeggildiana* has isodiametric epidermal cells with undulating or jagged anticlinal walls, whereas *S. paucipartita* possesses straight, well-defined anticlinal cell walls. Based on this cuticular feature, the Boserup specimens are best assigned to *Sphenobaiera paucipartita*.

Harris and Millington (1974) proposed a new genus, *Sphenarion*, designating *Sphenobaiera paucipartita* as the type species. However, we do not recognize sufficiently consistent morphological or microanatomical differences to warrant segregation of *Sphenarion* from *Sphenobaiera*.

Family. Ginkgoaceae

Genus. ***Sorosaccus*** T.M. Harris, 1935 emend. Liu *et al*., 2005

*Type species*. *Sorosaccus gracilis* T.M. Harris, 1935; basal Jurassic, East Greenland.

**Fig. 5.**
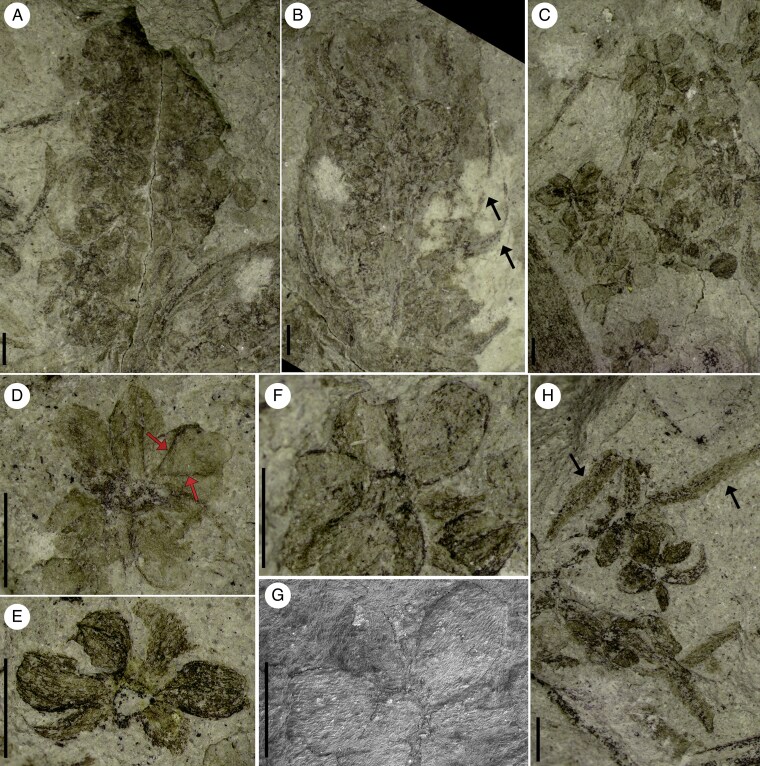
*Sorosaccus gracilis*. (A) Microsporangiate cone with numerous pollen sacs preserved on ill-defined sporophylls; PMU 32572. (B) Microsporangiate cone showing loosely arranged distally arched microsporophylls; PMU 32572. (C) Fragmentary microsporangiate organ with numerous pollen sacs attached in clusters to remnant sporophylls; PMU 32572. (D) Pollen sacs bearing longitudinal dehiscence slits (arrows); PMU 32591. (E) Longitudinally striate pollen sacs; PMU 32568. (F) Cluster of pollen sacs; PMU 32572. (G) SEM image of pollen sacs bearing fine longitudinal striae; PMU 32572. (H) Detached pollen sacs and linear microsporophylls; PMU 32560. Scale bar = 1 mm.

***Sorosaccus gracilis*** T.M. Harris, 1935 (Fig. 5A–H)

*Material*. Four specimens. PMU 32572, PMU 32591, PMU 32568, PMU 32560 (interval 9), Boserup beds.

*Description*. Several catkin-like microsporangiate structures (Fig. 5A–C) measuring >110 mm long and ∼7 mm wide, consisting of a central slender axis (0.75 mm wide) bearing helically arranged microsporophylls consisting of a proximal high-angled stub-like petiole and a distal strongly curved and acutely pointed lanceolate lamina (Fig. 5B, H; arrows). A cluster of pollen sacs is borne on the lateral and abaxial surface of the microsporophyll near the junction of the proximal petiole and lanceolate lamina. A few pollen sacs appear to be detached individually or as clusters of five to seven sacs (Fig. 5D–H). Each pollen sac is ovate, 0.8–1 mm long and 0.3–0.5 mm wide, with a rounded apex and bearing parallel longitudinal surface striae (Fig. 5E–G). No *in situ* pollen grains were recovered.

*Remarks*. Harris (1935) first described *Sorosaccus* from the *Thaumatopteris* Zone (basal Jurassic) in East Greenland. *Sorosaccus* is a fossil-genus defined as an elongate cylindrical male cone with a slender central rachis bearing thin, distally oriented, filiform sporangiophores measuring 2–5 mm long. The sporangiophores of the Greenland specimens bear clusters of approximately eight ovoid pollen sacs, which dehisce along a single longitudinal slit. The Boserup specimens are broadly consistent with the generic diagnosis provided by (Harris, 1935), particularly in having slender sporangiophores bearing microsporangial clusters with sacs that have longitudinal dehiscence slits (Fig. 5D).

Harris (1935) was uncertain about the affinities of *Sorosaccus* but suggested it might be affiliated with foliage of *Ginkgoites*, *Podozamites* or *Baiera*, with which it is co-preserved in the Greenland successions. Subsequent studies by Liu *et al*. (2005) and Zavialova *et al*. (2023) proposed that *Sorosaccus* represents a ginkgoalean microsporangiate organ. Liu *et al*. (2005) argued that *Sorosaccus gracilis* represents an early evolutionary step in ginkgoalean microsporangiate organ evolution bearing six to eight microsporangia per cluster, attached to the lateral and abaxial sides of a microsporophyll, whereas Early Cretaceous *Ginkgo liaoningensis* has three or four microsporangia, and extant *Ginkgo biloba* only two. This pattern suggests that *Sorosaccus* might have been part of a reduction series towards extant *Ginkgo* involving a decrease in the number of pollen sacs, shortening of the microsporophyll and loss of the curved lanceolate apex.

*Sorosaccu*s specimens from the Boserup beds occur in strata that are marginally older than the Greenland specimens, which Harris (1935) attributed to the Lower Jurassic *Thaumatopteris* Zone. Importantly, the new material was collected from interval 9 of the Boserup beds, where it co-occurs with leaves of *Czekanowskia* and *Ginkgoites*. Since *Czekanowskia* is typically affiliated with *Ixostrobus*-type microsporangiate organs with distinctive high-angled microsporophylls with terminal clusters of sporangia (Harris, 1935; Na *et al.*, 2021), this co-occurrence provides tentative support for affiliation with *Ginkgoites*, as proposed by Liu *et al.* (2005) and Zavialova *et al*. (2023).

Genus. ***Allicospermum*** T.M. Harris, 1935

*Type species*. *Allicospermum xystum* T.M. Harris, 1935; Upper Triassic, Greenland.

**Fig. 6.**
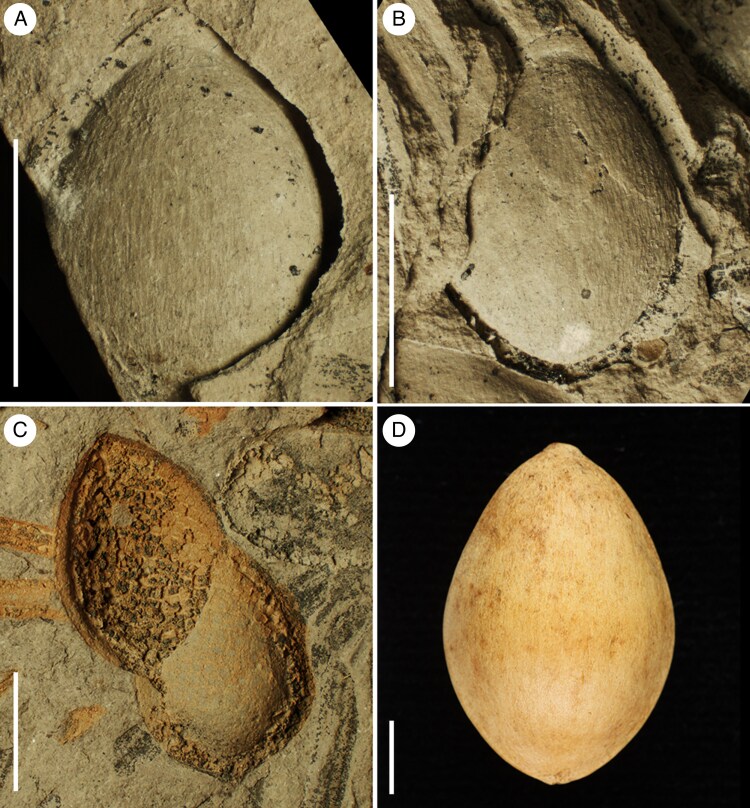
Moulds and internal casts of ovoid *Allicospermum* sp. seeds. (A) Internal cast of seed with an acute apex; PMU 32544. (B) Internal cast of seed with a sub-rounded chalaza; PMU 32547. (C) Top, external mould of seed retaining remnant organic matter with desiccation cracks. Bottom, seed representing an internal cast flanked by integument remains; PMU 32600. (D) Extant *Ginkgo biloba* L. seed with fleshy coating removed. Scale bar = 5 mm.

*Allicospermum* sp. (Fig. 6A–C)

*Material*. Three specimens. PMU 32544, PMU 32547, PMU 32600 (interval 9), Boserup beds.

*Description*. Moulds and internal casts of obovoid seeds, with an acute apex (Fig. 6A) and rounded chalaza (Fig. 6B), 7–9 mm long and 4.5–7 mm wide. The seeds have longitudinal parallel striations and desiccation cracks in 0.5- to 1-mm-thick remnant coalified integument tissue (Fig. 6C). The seeds have a slight longitudinal thickening or ridge imposing weak bilateral symmetry.

*Remarks*. These seeds are co-preserved with *Ginkgoites* leaves in interval 9 of the Boserup beds, suggesting affiliation with this foliage. The ovuliferous reproductive structures of ginkgoaleans have changed relatively little since the Jurassic (Zhou, 2009). The broadly similar morphologies of the seeds from the Boserup beds and those of modern *Ginkgo biloba* L. (Fig. 6D) also favour a ginkgoalean affinity for the Jurassic forms.

Other *Allicospermum*-type seeds co-occur with abundant *Ginkgo* leaves in the Middle Jurassic flora of Eriksdal, southern Sweden (Yang *et al.*, 2008), and in the Triassic–Jurassic floras of Scoresby Sound, Greenland (Harris, 1932; Tralau, 1966; Yang *et al.*, 2008). Those seeds have similar sizes and shape to the specimens described here.

Class. Pinopsida

Order. Pinales

Family. Cheirolepidiaceae Turutanova-Ketova, 1963

Genus. ***Brachyphyllum*** Brongniart, 1828

*Type species*. *Brachyphyllum mamillare* Brongniart 1828; Jurassic, Yorkshire, UK

**Fig. 7.**
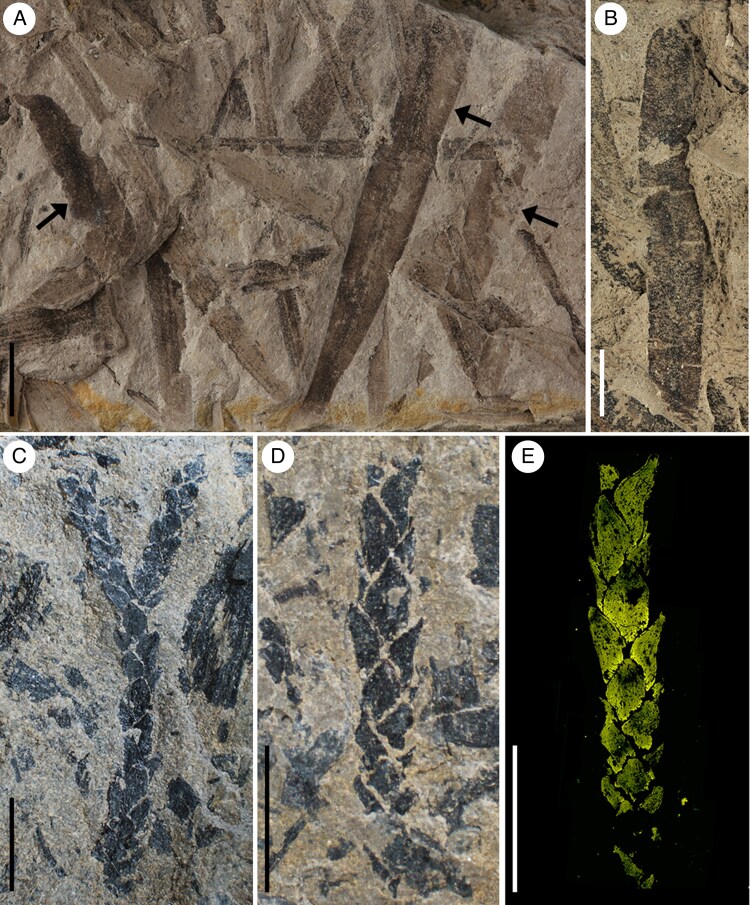
Conifer twig and leaf fragments. (A) Cluster of *Pityophyllum* sp. leaves with entire margins and prominent midvein; PMU 34549. (B) Detail of *Pityophyllum* sp. leaf with rounded apex; PMU 34541(b). (C) *Brachyphyllum* sp. cf. *B*. *crucis* leafy twig with distal bifurcation; PMU 34532(b). (D) *Brachyphyllum* sp. cf. *B*. *crucis* twig with spirally arranged, appressed, scale-like leaves with acute apices; PMU 34454. (E) *Brachyphyllum* sp. cf. *B*. *crucis* leafy twig photographed using fluorescence microscopy; PMU 34454. Scale bar = 5 mm.

**Fig. 8.**
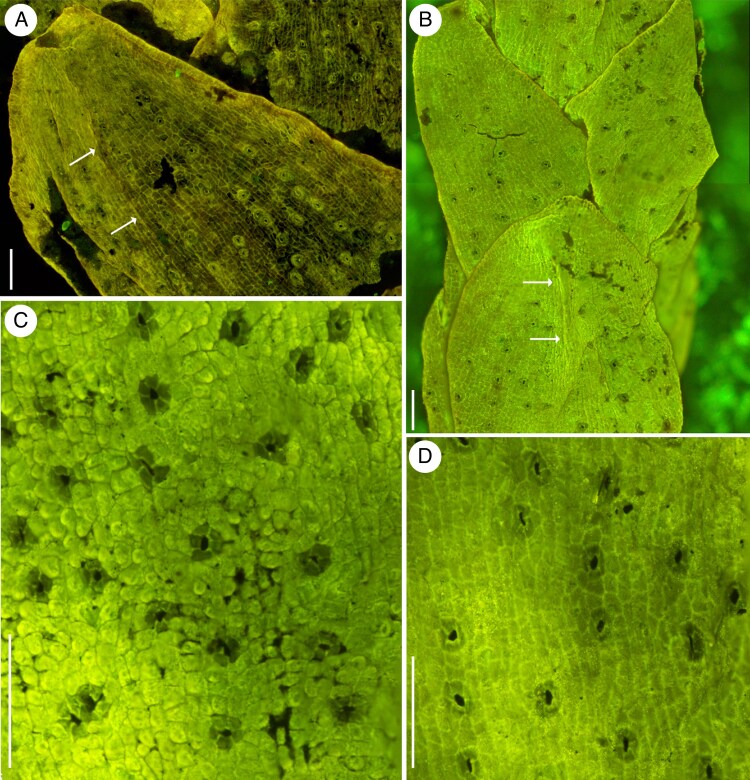
Epidermal cells and stomata of *Brachyphyllum* sp. cf. *B. crucis* photographed using fluorescence microscopy. (A) Abaxial stomata arranged in poorly defined longitudinal rows; PMU 34525. (B) Weakly keeled leaves with abaxial stomata in weakly defined rows or irregularly positioned; PMU 34496. (C) Abaxial leaf surface showing papillate epidermal cells and monocyclic stomata incorporating four to six subsidiary cells and locally surrounded by a ring of encircling cells; PMU 34496. (D) Abaxial leaf surface with rectangular to isodiametric polyhedral epidermal cells and stomata disposed in weakly defined longitudinal rows; PMU 34496. Arrows indicate weakly developed keel. Scale bar = 100 μm.

***Brachyphyllum* sp. cf. *B. crucis***, Kendall 1947 (Figs 7C–E and 8A–D)

*Material*. Four specimens. PMU 34525, PMU 34496, PMU 34532(b), PMU 34454 (interval 10), Boserup beds.

*Description*. Fragmentary axes, 12 mm long and 0.7–1.3 mm wide (Fig. 7C), with appressed rhombic–triangular weakly keeled, flattened, scale-like leaves up to 3 mm long and 0.6–2 mm wide (Fig. 8A, B). Leaves helically arranged, with pointed acute to bluntly obtuse apices (Fig. 7D, E). Leaf margins thin and membranous, fringed by a single row of transversely oriented rectangular cells (Fig. 8A, B). Leaves amphistomatic with stomata in weakly defined uniseriate rows on some leaves, but irregularly distributed on others, and sparse near leaf margins (Fig. 8C, D).

Guard cells in all stomata are sunken and surrounded by a ring of four to six subsidiary cells (Fig. 8C). Stomatal apparatus is monocyclic; subsidiary cells polygonal isodiametric and slightly sunken with respect to the epidermal cells; a row of encircling cells locally developed (Fig. 8C). Epidermal cells rectangular to isodiametric and polygonal in longitudinal rows (Fig. 8D). Subsidiary cells locally thickened adjacent to the stomatal pore but lacking pronounced papillae. Single papillae present on epidermal cells.

*Remarks*. The morphological characteristics of the specimens described here are consistent with *Brachyphyllum* (*sensu* T.M. Harris, 1979). *Pagiophyllum* leaves are proportionately longer than the near equidimensional leaves of *Brachyphyllum* (Kendall, 1948). Additionally, *Pagiophyllum* leaves are typically hypostomatic, whereas *Brachyphyllum* leaves are generally amphistomatic (Harris, 1979).

A comparative table of macro- and micromorphological diagnostic features of various *Brachyphyllum* species, presented by Batista *et al*. (2020), facilitated identification of the form from the Boserup beds. *Brachyphyllum comancheanum* Ash, from the Upper Jurassic of the USA, shares many macromorphological features with the *Brachyphyllum* leaves from the Boserup beds but differs in having marginal teeth and six to eight subsidiary cells, versus an entire margin and four to six subsidiary cells in the Boserup specimens. *Brachyphyllum pulcher* Lorch from the Lower Jurassic of Israel lacks a keeled leaf surface and papillae on the epidermal cells. The specimens from the Boserup beds are very similar to *Brachyphyllum mamillare* from the Jurassic of Yorkshire, in terms of branch arrangement, leaf shape, abaxial surface details, leaf apex and margin shape, stomatal arrangement, stomatal aperture, and subsidiary and epidermal cell shape. However, the leaves of *B. mamillare* typically have a high angle of insertion (Harris, 1979) and are known to be attached to microsporangiate cones bearing *Araucariacites*-type pollen (van Konijnenburg-van Cittert, 1971), indicating an araucariacean affinity. Another species known from the Yorkshire Middle Jurassic, *Brachyphyllum crucis* Kendall, has very similar leaves to *B. mamillare* but is associated with *Classopollis*-type pollen, a feature in common with the specimens from the Boserup beds. We initially discounted affiliation of the Swedish specimens with *B. crucis*, because Harris’s (1979) emended diagnosis of the species indicated that the leaves lack an abaxial keel, the stomata are not arranged in files and encircling cells are commonly developed around the subsidiaries. However, we note that some of the specimens illustrated by Harris (1979, Figs 6D, 7F) do possess keels, that the Boserup beds specimens have stomata variably aligned or scattered (Fig. 8A, B), and that rings of encircling cells are locally developed (Fig. 8C). On this basis, we consider the Swedish specimens to be closely comparable to *B. crucis* from Yorkshire, despite their minor age difference.

### Other plant remains

Several other plant remains occur in the assemblage but are generally too fragmentary or poorly preserved for confident identification even to genus level. Two samples (PMU 34549, PMU 34541(b) from interval 9; Fig. 7A, B) host isolated linear leaf fragments reaching 32 mm long and 2 mm wide with entire margins, gently contracted bases (Fig. 7A), rounded apices (Fig. 7B) and a prominent midvein. Although incomplete and lacking cuticular details, these leaves might be attributable to the conifer *Pityophyllum*. Chow (1924) described a specimen from Upper Triassic or Lower Jurassic strata of Skåne that reached 100 mm long.

**Fig. 9.**
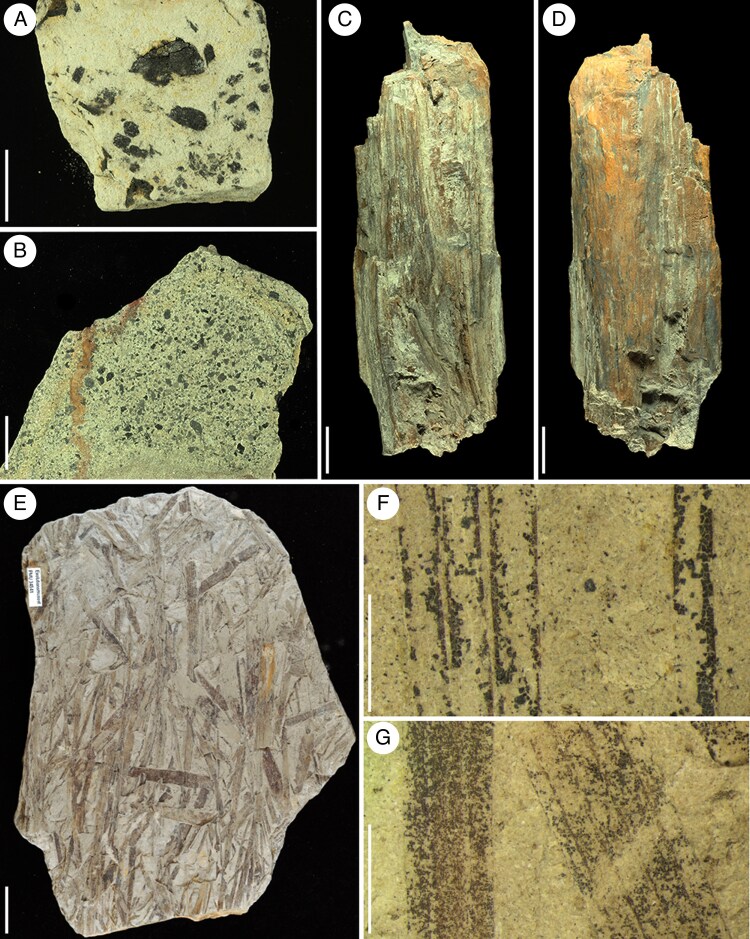
Plant remains from the Boserup beds. (A) Centimetre-scale charcoalified wood fragments in feldspathic sandstone; PMU 34540. (B) Millimetre-scale charcoalified plant fragments; PMU 34110. (C, D) Sideritized to weakly silicified woody axis in opposing views; PMU 35644. (E) Accumulation of isolated leaves representing a possible autumnal leaf mat; PMU 34541. (F) Coalified cuticle fragments of *Czekanowskia* sp.; PMU 32561 (G) Coalified plant fragments of *Pseudotorellia* sp.; PMU 32552. Scale bar = 2 cm. Modified from Vajda *et al*. (2023).

Additionally, remnants of charcoalified plant fragments occur in the interval 8 of the Boserup beds. These remains range from centimetre-scale (Fig. 9A) to millimetre-scale fragments (Fig. 9B), embedded in a fine- to medium-grained sandstone. Likewise, poorly permineralized (silicified and sideritized) wood remains, 180 mm long and 60 mm wide, are sparsely distributed in the same bed (Fig. 9C, D). The preservational states of these woods are inadequate for confident identification.

### Palynology

Interval 9, the ‘*Czekanowskia* bed’ (1.5 m thick) is devoid of pollen and spores. The palynofacies content comprises 94 % opaque phytoclasts and 6 % dispersed cuticle remains (Table 2).

**Fig. 10.**
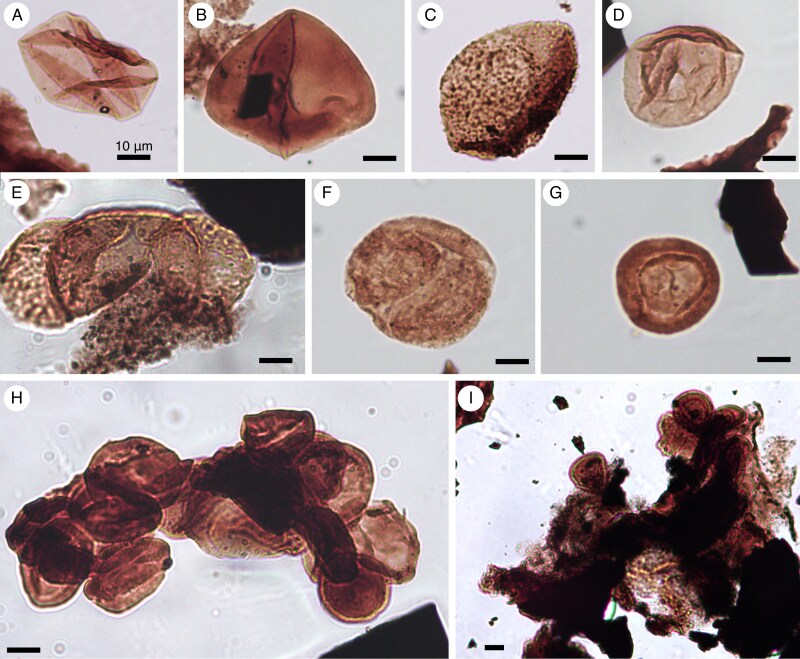
Representative spores and pollen from interval 10 (‘*Brachyphyllum* bed’) of the Boserup beds; PMU 34496. Specimen followed by England Finder Reference (EFR). (A) *Calamospora mesozoica*; EFR-F30. (B) *Cyathidites australis*; EFR-L41/1. (C) *Osmundacidites wellmanii*; EFR-F40/4. (D) *Aratrisporites minimus*; EFR- L40. (E, F) *Pinuspollenites minimus*; EFR-L41/4 and M41/2. (G) *Classopollis minor*; EFR-M42. (H) Cluster of *Classopollis minor*; EFR-Q17/2. (I) *Classopollis minor* partly still within pollen sac (microsporangium); EFR J29/2. Scale bar = 10 µm.

The sample from interval 10 ‘*Brachyphyllum* bed’ (3.5 m thick) contains a well-preserved but low-diversity palynoflora of 12 miospore taxa (seven spore taxa, five gymnosperm pollen taxa) and one freshwater alga (Fig. 10A–I; Table 1). No marine palynomorphs were identified in the assemblage. The dominant taxon is the cheirolepid conifer pollen *Classopollis minor* (Fig. 10G), which commonly occurs in aggregates (Fig. 10H) and, in some cases, partially intact pollen sacs (Fig. 10I). Additionally, sparse monosulcate pollen grains, potentially affiliated with *Sorosaccus* and *Ginkgoites* or *Czekanowskia*, are present. Spores constitute 24 % of the assemblage, and are represented mainly by smooth trilete spores (*Cyathidites*, Fig. 10B). The lycopsid spore *Aratrisporites minimus* (Fig. 10D) is also present in low relative abundance, accounting for around 7 % of the total palynoflora. The key taxon *Pinuspollenites minimus*, although present in low abundance (Fig. 10E, F) is indicative of a Hettangian age for interval 10 (Larsson, 2009). The palynofacies of the ‘*Brachyphyllum* bed’ (interval 10) consists of 88 % phytoclasts, of which 4 % represent opaque (black) phytoclasts and 84 % translucent (brown) phytoclasts; cuticle fragments constitute 11 % and pollen grains 1 % (Table 2). A significant proportion of the cuticles retain their epidermal cell patterning but are commonly broken and worn, suggesting significant transport.

## DISCUSSION

### Age of the Boserup beds flora of Skåne

All of the sampled beds are resolved to be of Hettangian age (Lower Jurassic) based on the composition of the recovered macroflora and the palynological assemblages. The sparse macrofloral assemblages from the lower part of the Boserup beds (intervals 8–10), including Equisitaleans, ginkgoopsids, cheirolepidiaceans and sparse ferns, signify a low-diversity post-extinction recovery flora. The poor preservation is due to transport in a high-energy fluvial system.

The productive palynoassemblage is dominated by *Classopollis* with lesser proportions of trilete spores, and the index taxa *Pinuspollenites minimus* and *Calamospora tener*, all indicative of an earliest Jurassic age. No typical Rhaetian taxa, such as *Ricciisporites tuberculatus*, *Rhaetipollis germanicus* or *Ovalipollis* spp., were identified. The palynoassemblage is attributable to Dybkjaer’s (1988) zone B, Michelsen's (1979) zone C_1_ and Lund’s (1977)*Pinuspollenites–Trachysporites* Zone, all considered Hettangian in age.

Previous studies (Lindström and Erlström, 2006) identified mixed Rhaetian–Hettangian assemblages from sandstone samples in the basal 2 m of the Boserup beds at Norra Albert (equivalent to interval 8 herein). Whereas pollen and spores are more resilient to transport than leaves, they are prone to reworking in high-energy sedimentary systems and, in this context, can be less reliable as biostratigraphic indices in the sandstones.

### The vegetation succession

The lowermost occurrences of plant remains within the Boserup beds are at the very base of the Jurassic succession (interval 8; Fig. 1D), where fragments of parallel-veined leaves tentatively referred to *Sphenobaiera paucipartita* (Fig. 4A, I) were recovered along with a single occurrence of *Neocalamites* sp. (Fig. 2A).

The plant assemblage from interval 9 (at ∼5 m above the base of the Boserup beds) is characterized by ginkgoopsids, including *Czekanowskia* sp., *Ginkgoites* sp. cf. *G*. *marginatus* and *Pseudotorellia* sp., with sparse representation of linear univeined *Pityophyllum* leaves. The lack of palynomorphs, the presence of corroded cuticle remains and the fragmentation of leaves suggest transport over moderate distances and subsequent deposition in a relatively high-energy environment.

The uppermost plant assemblage from interval 10 (∼8 m above the base of the Boserup beds) is dominated by *Brachyphyllum* sp. cf. *B*. *crucis*, presumably forming the canopy, and an understorey of ferns (*Cladophlebis* spp.) with sparse sphenophytes (*Neocalamites* sp.) that likely grew on the margins of water bodies. *Classopollis* pollen is known to be produced by Cheirolepidiaceae (Hluštík and Konzalova, 1976; Alvin, 1982; Kva”ek, 2000). Its abundance as isolated grains and clusters in association with *Brachyphyllum* sp. cf. *B*. *crucis*, the only conifer foliage in interval 10, suggests a biological link between these remains. Smooth trilete spores in this interval were possibly affiliated with dipteridaceous ferns, although no macrofossils of this group have yet been identified. The sparse *Neocalamites* axes within this interval are consistent with the low abundance of sphenopsid spores.

Although well-preserved *Classopollis* pollen (some *in situ* in sacs) was recovered from interval 10, the abraded phytoclasts, poorly preserved cuticles and fragmented fern pinnae and conifer branches indicate that this assemblage was subject to moderate transport. Hence, the palynoflora might include contributions from some plants growing considerable distances from the depositional site.

The occurrence of dispersed charcoalified plant remains (Fig. 9A, B) 3 m above the base of the Boserup beds (uppermost part of interval 8; Fig. 1D) at Norra Albert suggests that wildfires impacted the post-ETE landscape of southern Sweden. This is consistent with previous results from Europe and Greenland showing that forest fires and deforestation were a significant feature of the regional latest Triassic and Hettangian landscape (McElwain *et al.*, 2007; Marynowski and Simoneit, 2009; van de Schootbrugge *et al.*, 2009; Belcher *et al.*, 2010; Petersen and Lindström, 2012; Vajda *et al.*, 2016).

### Comparison with coeval post-recovery floras

Palaeo-CO_2_ estimations using the stomatal-based proxy method applied to Triassic and Jurassic fossil cuticles from regions such as Greenland, Sweden, Germany, Northern Ireland and China confirm the increase in CO_2_ levels, rising from around 600 ppm before the ETE event to >2000 ppm during the Hettangian (McElwain, 2018). Moreover, the release of >8000 Gt of CO_2_ to the atmosphere is argued to have produced global warming, marine anoxia leading to algal blooms, and disruptions to the carbon cycle (van de Schootbrugge *et al.*, 2009). However, it remains unclear how these climatic perturbations impacted terrestrial vegetation across different regions during the Triassic–Jurassic transition. Macrofloral evidence from Triassic–Jurassic successions of East Greenland and palynological records from the Newark Basin, in eastern North America, indicate major taxon losses and turnover of plant communities (Fowell *et al.*, 1994; McElwain *et al.*, 2007), whereas macrofossil floras from certain regions in Europe indicate only minor changes in terrestrial plant composition (Barbacka *et al.*, 2017).

Because the terrestrial vegetation response to the end-Triassic crisis appears to have varied geographically, investigating Early Jurassic floras across the region provides critical insights into how plant communities recovered under stressed environmental conditions. Early Jurassic floras are known from the Pliensbachian of Bornholm, Denmark (Mehlqvist *et al.*, 2009), Sinemurian–Pliensbachian of Chmielów, Holy Cross Mountains, Poland (Harris, 1961), Sinemurian–Pliensbachian of Veneto, northern Italy (Wesley, 1965, 1974; Bartiromo and Lumaga, 2009), Hettangian–Sinemurian of the Mecsek Mountains, Hungary (Barbacka, 2011) and the Hettangian of Franconia, Germany (Weber, 1968; Van Konijnenburg-van Cittert *et al.*, 2021). Here, we compare the Boserup beds assemblages only with earliest Hettangian recovery floras from similar palaeolatitudes, namely those from the Odrowąż (Sołtyków) site, the Lower Gromadzice site and the Huta borehole (Zagaje Formation, Poland; Pacyna, 2013; Pacyna *et al.*, 2022), and the lowermost 25 m of the *Thaumatopteris* Zone in Scoresby Sound, East Greenland (Harris, 1937) (Supplementary Data Table S1).

The Boserup beds flora, although comparatively modest in size, shares sphenophytes with Poland. Fern diversity in the Boserup beds appears limited to *Cladophlebis*, whereas floras from East Greenland and Poland host a broader representation, including *Dictyophyllum*, *Phlebopteris*, *Todites* and *Thaumatopteris* species. This low fern diversity at Norra Albert may reflect poor fossil preservation, limited sampling or genuinely low richness. Caytoniales and Bennettitales are already present in the earliest Hettangian floras of East Greenland and Poland but are notably absent in the immediate post-extinction strata of the Boserup beds. Nevertheless, Bennettitales subsequently appear in the later Hettangian of Skåne (Pott and McLoughlin, 2009). Importantly, Ginkgoales and Czekanowskiales are consistently documented across all earliest Hettangian floras examined (Poland, East Greenland and Sweden), suggesting their ecological resilience and capacity for recovery following the end-Triassic extinction. In terms of conifer diversity, Poland hosts a richer assemblage (*Hirmeriella*, *Podozamites*, *Schizolepis*, *Stachyotaxus*) compared with the more depleted floras of the Boserup beds (*Brachyphyllum*, *Pseudotorellia*) and East Greenland (*Swedenborgia*). In particular, interval 9 of the Boserup beds hosts an assemblage (Fig. 9E) strongly resembling that of the upper part of the Zagaje Formation, dated as latest early Hettangian (Pacyna *et al.*, 2022). Both assemblages are dominated by ginkgoaleans (*Sphenobaiera*), czekanowskialeans (*Czekanowskia*) and *Desmiophyllum* (possible conifers), interpreted here as *Ginkgoites* sp. cf. *G*. *marginatus* and *Pseudotorellia* sp. Conifers form the second most abundant group in both assemblages, represented in Poland by *Podozamites*, *Pityophyllum* and rare *Brachyphyllum*, whereas in the Boserup beds *Brachyphyllum* is the dominant conifer taxon, followed by sparse *Pityophyllum*.

Expanding the comparison beyond these northern European sites, floras from central Europe (lowermost Hettangian of Poland, Hettangian of Germany and Hettangian–Sinemurian of Hungary) reveal the shared presence of *Nilssonia* (Supplementary Data Table S1) but its absence from the earliest Hettangian floras of East Greenland and from the Boserup beds. Additionally, ferns, Caytoniales, cycads, Ginkgoales and conifers appear to be more diverse in the central European floras of Poland, Hungary and Germany (Supplementary Data Table S1).

Czekanowskiales are consistently recorded in the earliest Hettangian floras of Sweden, Poland and East Greenland, which aligns with their abundance in high-palaeolatitude Siberian deposits, where they are recognized as a pioneer riparian plant group that was successionally replaced by *Phoenicopsis*-dominated forests (Krassilov, 2003).

The limited diversity observed in the Boserup beds flora likely reflects a genuine signal of slow vegetation recovery following the ETE, as indicated by the presence of pioneer taxa, such as sphenophytes, ferns and czekanowskialeans, and the initial absence of seed-plant groups typical of northern European climax community assemblages (e.g. Bennettitales, Caytoniales and Cycadales). Although this ecological signal may have been partially overprinted by taphonomic biases related to fossil preservation and by limited sampling, the overall floral composition suggests a biological response (depauperate diversity and preponderance of opportunist taxa) to the stressed conditions during the extinction event.

### Cheirolepids from the Boserup beds align with the Early Jurassic global spike

Cheirolepidiaceae is an extinct group of Mesozoic conifers spanning the Late Triassic to the Late Cretaceous (Jin *et al.*, 2023; Marmi *et al.*, 2023). The current fossil evidence of this group is diverse, including foliage, cones, wood and typical *Classopollis*-type pollen (Alvin, 1982; Watson, 1988; Taylor *et al.*, 2009; Mendes and Kva”ek, 2022; Jin *et al.*, 2023). Cheirolepids had varied statures (shrubs to trees) and occupied diverse habitats, ranging from subtropical–tropical forests (Alvin, 1982; Wang *et al*., 2005*b*), dry and saline coastal environments (Kva”ek, 2000; McArthur *et al.*, 2016; Escapa and Leslie, 2017; Slater *et al.*, 2017), freshwater coastal habitats (Marmi *et al.*, 2023) and upland settings (Petersen and Lindström, 2012) to humid lowland forests of southern high latitudes (Tosolini *et al.*, 2015). European mid- to late Mesozoic species with appressed scale-like leaves (e.g. *Frenelopsis* and *Pseudofrenelopsis*) were generally xerophilous (Mendes and Kvaček, 2022; Kvaček *et al.*, 2025) with particular adaptations to periods of severe water stress (Mendes *et al.*, 2023).The cheirolepid occurrences (*Brachyphyllum*) from the Boserup beds are consistent with global Early Jurassic vegetation patterns, whereby high relative abundances of *Classopollis* (Cheirolepidiaceae) have been recorded from coeval Swedish localities (Lund, 1977; Vajda, 2001; Larsson, 2009; Vajda *et al.*, 2013; Peterffy *et al.*, 2016), Greenland (Pedersen and Lund, 1980) and Britain (Mander *et al.*, 2013). As far afield as eastern Australia, Cheirolepidiaceae pollen is common in Hettangian and Sinemurian (Lower Jurassic) assemblages (Helby *et al.*, 1987; Jansson *et al.*, 2008; de Jersey and McKellar, 2013). A similar abundance of cheirolepidiaceans has been observed in Lower Jurassic assemblages of the Sichuan Basin, southern China (Li *et al.*, 2020). Lower Jurassic successions in Asturias, Spain, are also dominated by cheirolepidiaceous conifers but occur in association with Araucariaceae, Pinaceae and Ginkgoales/Cycadales/Bennettitales, as inferred from palynofloral assemblages (Diéguez *et al.*, 2010). In the Newark Basin, North America, a fern spike identified in basal Jurassic strata is immediately succeeded by the dominance of *Classopollis meyeriana* (Olsen *et al.*, 2002; Whiteside *et al.*, 2007). Cheirolepids proliferated rapidly in the Early Jurassic, likely filling the ecological niches previously occupied by various ‘seed-fern’ groups of the Late Triassic (Vajda and Bercovici, 2014) and the pulse in *Classopollis* abundance is generally used as a global marker for basal Jurassic strata.

### Significance of *Czekanowskia*

*Czekanowskia* is a common plant in Mesozoic floras, and is characterized by elongate, highly dissected slender leaves (Fig. 3A, B; Harris, 1935; Taylor *et al.*, 2009). It ranges from the Upper Triassic to Upper Cretaceous, being common and broadly distributed in Lower–Middle Jurassic strata across the Northern Hemisphere (Harris and Miller, 1974; Vakhrameev, 1987; Samylina and Kiritchkova, 1993; Ash, 1994; Sun *et al.*, 2009, 2015). This group experienced a significant decline, probably due to large-scale transgression from northern seas and expansion of arid climates, becoming almost extinct at the end of the Jurassic with regionally restricted records from the Cretaceous (Kimura and Okawara, 1982; Ash, 1994; Sun *et al*., 2001).

In Sweden, *Czekanowskia rigida* was first reported by Nathorst (1886) in the Rhaetian (Late Triassic) flora of Bjuv, based on only a single leaf sample, which was later renamed as *Czekanowskia nathorstii* by Harris (1935). To our knowledge, no further Czekanowskiales have been documented from Upper Triassic strata of Sweden, whereas several Hettangian (Early Jurassic) *Czekanowskia* occurrences were reported from the Helsingborg Member at Stabbarp coal mine and from the Höör Sandstone of central Skåne (Nathorst, 1906; Antevs, 1919; Johansson, 1922). The presence of this plant group in the Boserup beds and in coeval deposits of Poland (Pacyna, 2013; Pacyna *et al.*, 2022) and East Greenland (McElwain *et al.*, 2007; Bacon *et al.*, 2011), where it reaches relative abundances of 52 %, highlight this taxon as an important component of the regional early Hettangian (Early Jurassic) vegetation.

### Palaeoclimate signature

The climatic signature of the collective Boserup beds flora is ambiguous. The assemblage from interval 9 is very similar to coeval floras from various parts of Greenland, central Europe and Asia. The mix of needle-leafed conifer and ginkgoalean and czekanowskialean remains suggests affiliation with humid cool-temperate conditions. The dominant taxon, *Czekanowskia*, was distributed mainly from the humid cool temperate Northern Hemisphere regions of Siberia, Inner Mongolia and northern China to the humid subtropical regions near the Tethys Ocean and southern China (Ash, 1994; Sun *et al*., 2009, 2015) during the Mesozoic. Lower Jurassic ginkgoaleans have been reported from multiple deposits of presumed humid temperate settings in the North Atlantic–European province, such as the Zagaje and Skłoby formations in southern Poland, in which *Czekanowskia rigida*, ‘*Ginkgo*’ sp. aff. *G. whittbyensis*, ‘*Ginkgo*’ *sibirica* and *Ginkgoites taeniata* are co-preserved (Makarewiczówna, 1928; Pacyna, 2013); the Mecsek Coal Formation in southern Hungary, where the most abundant taxa are *Ginkgoites marginatus* and *Baiera furcata* (Barbacka, 2011); the Steierdorf Formation of southwestern Romania hosting *Ginkgoites marginatus* (Barbacka *et al.*, 2016); and the post-extinction flora of Astartekløft, East Greenland, in which *Sphenobaiera* and *Ginkgoites* are subdominant after *Czekanowskia* (McElwain *et al.*, 2007).

Cheirolepid conifers are generally interpreted as drought-resistant taxa in European, Asian and in some Southern Hemisphere Jurassic–Cretaceous assemblages (Alvin, 1982; Watson, 1988; Wang *et al*., 2005*b*; Jansson *et al.*, 2008), superficially suggesting warmer and drier conditions for interval 10 than the climatic signature of the flora represented in interval 9. However, this group had a broader climatic envelope in the Mesozoic than formerly supposed (Watson, 1988; Tosolini *et al*., 2015) and, in the absence of heavy stomatal protection, some representatives likely occupied humid and cool-temperate settings.

### Conclusions

Our palaeobotanical and palynological study of the Boserup beds, Skåne, Sweden, shows that the succession hosts sparse but important, typical Lower Jurassic macrofloral and palynological assemblages that provide a glimpse into the recovery flora following the end-Triassic mass extinction in a middle–high latitude setting. This pioneer flora, dominated by Ginkgoopsida and cheirolepid conifers (well preserved *Brachyphyllum* and abundant *Classopollis minor*), has features in common with coeval recovery ecosystems documented from Greenland, Poland and China, but also from the Southern Hemisphere (Australian and New Zealand) successions. Further studies on larger sample sets combined with high-precision radioisotopic dating will be needed to answer whether these floras represented a global surge in pioneer ginkgoopsid and cheirolepids forests or if their appearance and high abundance is diachronous across palaeolatitudes and, rather, was tied to successive climate change. Additional comparisons with the lower and middle parts of the Zagaje Formation in the Holy Cross Mountains of Poland will be necessary, as both areas represent marginal settings of the same sedimentary basin.

## Supplementary Material

mcaf143_Supplementary_Data

## SUPPLEMENTARY DATA

Supplementary data are available at *Annals of Botany*online and consist of the following. Table S1. Comparison of Boserup beds flora with Earliest Hettangian and Hettangian–Sinemurian assemblages from East Greenland, Poland, Hungary and Germany.

## References

[mcaf143-B1] Ahlberg A , Sivhed U, Erlström M. 2003. The Jurassic of Skåne, southern Sweden. Geological Survey of Denmark and Greenland Bulletin 1: 527–541.

[mcaf143-B2] Alvin KL . 1982. Cheirolepidiaceae: biology, structure and paleoecology. Review of Palaeobotany and Palynology 37: 71–98.

[mcaf143-B3] Anderson JM , Anderson HM, Cleal CJ. 2007. Brief history of the gymnosperms: classification, biodiversity, phytogeography and ecology. Pretoria: South African National Biodiversity Institute.

[mcaf143-B4] Antevs E . 1919. Die Liassische flora des Hörsandsteins. Kungliga Svenska Vetenskapsakademiens Handlingar 59: 1–71.

[mcaf143-B5] Ash S . 1994. First occurrence of *Czekanowskia* (Gymnospermae, Czekanowskiales) in the United States. Review of Palaeobotany and Palynology 81: 129–140.

[mcaf143-B6] Bacon KL , Belcher CM, Hesselbo SP, McElwain JC. 2011. The Triassic–Jurassic boundary carbon-isotope excursions expressed in taxonomically identified leaf cuticles. PALAIOS 26: 461–469.

[mcaf143-B7] Barbacka M . 2011. Biodiversity and the reconstruction of early Jurassic flora from the Mecsek mountains (southern Hungary). Acta Palaeobotanica 51: 127–179.

[mcaf143-B8] Barbacka M , Bodor E, Jarzynka A, et al 2014a. European Jurassic floras: statistics and palaeoenvironmental proxies. Acta Palaeobotanica 54: 173–195.

[mcaf143-B9] Barbacka M , Pacyna G, Feldman-Olszewska A, Ziaja J, Bodor E. 2014b. Triassic-Jurassic flora of Poland; floristical support of climatic changes. Acta Geologica Polonica 64: 281–309.

[mcaf143-B10] Barbacka M , Pacyna G, Kocsis”T, Jarzynka A, Ziaja J, Bodor E. 2017. Changes in terrestrial floras at the Triassic-Jurassic boundary in Europe. Palaeogeography, Palaeoclimatology, Palaeoecology 480: 80–93.

[mcaf143-B11] Barbacka M , Popa ME, Mitka J, Bodor E, Püspöki Z, McIntosh RW. 2016. A quantitative approach for identifying plant ecogroups in the Romanian early Jurassic terrestrial vegetation. Palaeogeography, Palaeoclimatology, Palaeoecology 446: 44–54.

[mcaf143-B12] Bartiromo A , Lumaga MRB. 2009. Taxonomical revision of the collection of Jurassic plants from Roverè di Velo (Veneto, northern Italy) stored in the palaeontological museum of the University of Naples “Federico II”. Bollettino Della Societa Paleontologica Italiana: Societa Paleontologica Italiana 48: 1–13.

[mcaf143-B13] Batista MEP , Kunzmann L, Sá AA, Saraiva A”F, Loiola MIB. 2020. A new species of *Brachyphyllum* from the Crato formation (Lower Cretaceous), Araripe Basin, Brazil. Ameghiniana 57: 519–533.

[mcaf143-B14] Bauer K , Kustatscher E, Krings M. 2013. The ginkgophytes from the German Kupferschiefer (Permian), with considerations on the taxonomic history and use of *Baiera* and *Sphenobaiera*. Bulletin of Geosciences 88: 539–556.

[mcaf143-B15] Belcher CM , Mander L, Rein G, et al 2010. Increased fire activity at the Triassic/Jurassic boundary in Greenland due to climate-driven floral change. Nature Geoscience 3: 426–429.

[mcaf143-B16] Bomfleur B , Blomenkemper P, Kerp H, McLoughlin S. 2018. Polar regions of the Mesozoic–Paleogene greenhouse world as refugia for relict plant groups. In: Krings M, Harper CJ, Cúneo NR, Rothwell GW. eds. Transformative paleobotany: papers to commemorate the life and legacy of Thomas N. Taylor. Amsterdam: Elsevier, 593–611.

[mcaf143-B17] Bos R , Lindström S, van Konijnenburg-van Cittert H, et al 2023. Triassic-Jurassic vegetation response to carbon cycle perturbations and climate change. Global and Planetary Change 228: 104211.

[mcaf143-B18] Brongniart AT . 1849. Tableau des genres de végétaux fossiles considérés sous le point de vue de leur classification botanique et de leur distribution géologique. Dictionnaire Universel d’Histoire Naturelle.

[mcaf143-B19] Capriolo M , Marzoli A, Aradi LE, et al 2020. Deep CO2 in the end-Triassic central Atlantic Magmatic Province. Nature Communications 11: 1670. 10.1038/s41467-020-15325-6 PMC7138847 32265448

[mcaf143-B20] Chow TC . 1924. The lower Liassic flora of Sofiero and Dompäng in Scania. Arkiv för Botanik 19: 1–19.

[mcaf143-B21] de Jersey NJ , McKellar JL. 2013. The palynology of the Triassic–Jurassic transition in southeastern Queensland, Australia, and correlation with New Zealand. Palynology 37: 77–114.

[mcaf143-B22] Diéguez C , Peyrot D, Barrón E. 2010. Floristic and vegetational changes in the Iberian Peninsula during Jurassic and Cretaceous. Review of Palaeobotany and Palynology 162: 325–340.

[mcaf143-B23] Dybkjær K . 1988. Palynological zonation and stratigraphy of the Jurassic section in the Gassum No. 1-borehole, Denmark. Danmarks Geologiske Undersøgelse, DGU serie A 21: 1–73.

[mcaf143-B24] Elgorriaga A , Escapa IH, Cúneo NR. 2019. Relictual *Lepidopteris* (Peltaspermales) from the early Jurassic Cañadón Asfalto formation, Patagonia, Argentina. International Journal of Plant Sciences 180: 578–596.

[mcaf143-B25] Engler A , Prantl K. 1897. Die Natürlichen Pflanzenfamilien nebst ihren Gattungen und wichtigeren Arten, insbesondere den Nutzpflanzen, unter Mitwirkung zahlreicher hervorragender Fachgelehrten. Leipzig: W. Engelmann.

[mcaf143-B26] Escapa I , Leslie A. 2017. A new Cheirolepidiaceae (Coniferales) from the early Jurassic of Patagonia (Argentina): reconciling the records of impression and permineralized fossils. American Journal of Botany 104: 322–334. 28213347 10.3732/ajb.1600321

[mcaf143-B27] Fowell SJ , Cornet B, Olsen PE. 1994. Geologically rapid late Triassic extinctions: palynological evidence from the Newark supergroup. Geological Society of America Special Paper 288: 197–206.

[mcaf143-B28] Göppert HR . 1846. Über die fossile Flora der mittleren Juraschichten in Oberschlesien. Uebersicht der Arbeiten und Veränderungen der Schlesischen Gesellschaft für vaterländische Cultur im Jahre, 1845.

[mcaf143-B29] Gorozhankin IN . 1904. Lektsii po morfologii i sistematike archegonialnykh rastenij. Vol. II. Moskva: Mamontov.

[mcaf143-B30] Guy-Ohlson D , Norling E. 1994. Jurassic sequences in Sweden. Geobios 17: 275–286.

[mcaf143-B31] Halle TG . 1908. Zur Kenntnis der mesozoischen Equisetales Schwedens. Kungliga Svenska Vetenskapsakademiens Handlingar 43: 1–56.

[mcaf143-B32] Harris TM . 1931. The fossil flora of Scoresby Sound east Greenland, part 1: cryptogams (exclusive of Lycopodiales). Meddelelserom Grønland 85: 1–104.

[mcaf143-B33] Harris TM . 1932. The fossil flora of Scoresby Sound, east Greenland: part 2: description of seed plants incertae sedis together with a discussion of certain cycadophyte cuticles. Meddelelser om Grønland 85: 1–112.

[mcaf143-B34] Harris TM . 1935. The fossil flora of Scoresby Sound, east Greenland. Part 4: Ginkgoales, Coniferales, Lycopodiales and isolated fructifications. Meddelelser om Grønland 112: 1–176.

[mcaf143-B35] Harris TM . 1937. The fossil flora of Scoresby Sound, east Greenland. Part 5. Stratigraphic relations of the plant beds. Meddelelser om Grønland 112: 1–114.

[mcaf143-B36] Harris TM . 1961. The Yorkshire Jurassic flora. I. Thallophyta-Pteridophyta. London: Trustees of the British Museum (Natural History).

[mcaf143-B37] Harris TM . 1979. The Yorkshire Jurassic flora. V. Coniferales. London: Trustees of the British Museum (Natural History).

[mcaf143-B38] Harris TM , Miller J. 1974. The Yorkshire Jurassic flora. IV. Czekanowskiales. London: Trustees of the British Museum (Natural History).

[mcaf143-B39] Harris TM , Millington W. 1974. The Yorkshire Jurassic Flora. IV. Ginkgoales. London: Trustees of the British Museum (Natural History).

[mcaf143-B40] Helby R , Morgan R, Partridge A. 1987. A palynological zonation of the Australian Mesozoic. Memoir of the Association of Australasian Palaeontologists 4: 1–94.

[mcaf143-B41] Hluštík A , Konzalova M. 1976. *Frenelopsis alata* (K. Feistm.) Knobloch (Cupressaceae) from the Cenomanian of Bohemia, a new plant producing *Classopollis* pollen. In: *Evolutionary Biology, Proceedings of Conference, 1975, Liblice*, 125–131.

[mcaf143-B42] Jansson IM , McLoughlin S, Vajda V, Pole M. 2008. An early Jurassic flora from the Clarence-Moreton Basin, Australia. Review of Palaeobotany and Palynology 150: 5–21.

[mcaf143-B43] Jin P , Zhang M, Du B, Li A, Sun B. 2023. A new species of *Pararaucaria* from the lower Cretaceous of Shandong province (Eastern China): insights into the evolution of the Cheirolepidiaceae cone. Cretaceous Research 146: 105475.

[mcaf143-B44] Johansson N . 1922. Die rätische Flora der Kohlengruben bei Stabbarp und Skromberga in Schonen. Kungliga Svenska Vetenskapsakademiens Handlingar 63: 1–78.

[mcaf143-B45] Kendall MW . 1948. On six species of *Pagiophyllum* from the Jurassic of Yorkshire and southern England. Annals and Magazine of Natural History 1: 73–108.

[mcaf143-B46] Kimura T , Okawara H. 1982. *Solenites* sp. (Czekanowskiales) from the upper Cretaceous Omichidani formation, in the inner zone of Southwest Japan. Proceedings of the Japan Academy, Series B 58: 204–207.

[mcaf143-B47] Krassilov VA . 1972. Mesozoic Flora of the Bureya River (Ginkgoales and Czekanowskiales) [in Russian]. Moscow: Nauka.

[mcaf143-B48] Krassilov VA . 2003. Terrestrial palaeoecology and global change. Sofia: Pensoft.

[mcaf143-B49] Krüger A , Slater S, Vajda V. 2021. 3D imaging of shark egg cases (*Palaeoxyris*) from Sweden with new insights into early Jurassic shark ecology. Geologiska Föreningen i Stockholm Förhandlingar 143: 229–247.

[mcaf143-B50] Kustatscher E , van Konijnenburg-van Cittert J. 2013. Seed ferns from the European Triassic – an overview. In: The Triassic system: new developments in stratigraphy and paleontology. Vol. 61. New Mexico Museum of Natural History and Science, 331–344.

[mcaf143-B51] Kvaček J . 2000. *Frenelopsis alata* and its microsporangiate and ovuliferous reproductive structures from the Cenomanian of Bohemia (Czech Republic. Central Europe). Review of Palaeobotany and Palynology 112: 51–78. 11042326 10.1016/s0034-6667(00)00035-x

[mcaf143-B52] Kvaček J , Čerňanský A. 2024. Early Cretaceous *Equisetites* from Slovakia. Palaeobiodiversity and Palaeoenvironments 104: 237–243.

[mcaf143-B53] Kvaček J , Mendes MM, Van Konijnenburg-van Cittert JHA. 2025. *Frenelopsis callapezii*, a new Cheirolepidiaceous conifer from the lower Cretaceous (Upper Aptian–Lower Albian) sedimentary deposits of Lusitanian Basin in Western Portugal: systematic and palaeoenvironmental implications. International Journal of Plant Sciences 186: 178–192.

[mcaf143-B54] Landwehrs J , Feulner G, Petri S, Sames B, Wagreich M. 2021. Investigating Mesozoic climate trends and sensitivities with a large ensemble of climate model simulations. Paleoceanography and Paleoclimatology 36: e2020PA004134. 10.1029/2020PA004134 PMC8251552 34240008

[mcaf143-B55] Larsson LM . 2009. Palynostratigraphy of the Triassic–Jurassic transition in southern Sweden. Geologiska Föreningen i Stockholm Förhandlingar 131: 147–163.

[mcaf143-B56] Li L , Wang Y, Kürschner WM, Ruhl M, Vajda V. 2020. Palaeovegetation and palaeoclimate changes across the Triassic–Jurassic transition in the Sichuan Basin, China. Palaeogeography, Palaeoclimatology, Palaeoecology 556: 109891.

[mcaf143-B57] Li L , Wang Y, Liu Z, Zhou N, Wang Y. 2016. Late Triassic palaeoclimate and palaeoecosystem variations inferred by palynological record in the northeastern Sichuan Basin, China. Palaontologische Zeitschrift 90: 327–348.

[mcaf143-B58] Lindström S , Erlström M. 2006. The late Rhaetian transgression in southern Sweden: regional (and global) recognition and relation to the Triassic–Jurassic boundary. Palaeogeography, Palaeoclimatology, Palaeoecology 241: 339–372.

[mcaf143-B59] Lindström S , Erlström M, Piasecki S, Nielsen LH, Mathiesen A. 2017. Palynology and terrestrial ecosystem change of the middle Triassic to lowermost Jurassic succession of the eastern Danish basin. Review of Palaeobotany and Palynology 244: 65–95.

[mcaf143-B60] Liu X-Q , Hueber FM, Li C-S, Wang Y-F. 2005. Emendation of *Sorosaccus gracilis* Harris 1935, a gymnospermous pollen cone. Journal of Systematics and Evolution 43: 182–190.

[mcaf143-B61] Lund JJ . 1977. Rhaetic to lower Liassic palynology of the onshore south-eastern North Sea basin. Danmarks Geologiske Undersøgelse II. Række 109: 1–128.

[mcaf143-B62] Lundblad B . 1950. Studies in the Rhaeto-Liassic floras of Sweden: Pteridophyta, Pteridaspermae, and Cycadophyta from the mining district of NW Scania. Kungliga Svenska Vetenskapsakademiens Handlingar 1: 1–82.

[mcaf143-B63] Lundblad B . 1959a. Rhaeto-Liassic floras and their bearing on the stratigraphy of Triassic-Jurassic rocks. Stockholm Contributions in Geology 3: 83–102.

[mcaf143-B64] Lundblad B . 1959b. Studies in the Rhaeto-Liassic floras of Sweden. II: Ginkgophyta from the mining district of NW Scania. *Kungliga Svenska Vetenskapsakademiens Handlingar*. Fjärde Serien 6: 1–38.

[mcaf143-B65] Makarewiczówna A . 1928. Flora dolno-liasowa okolic Ostrowca. Prace Towarzystwa Przyjaciół Nauk w Wilnie, Wydział Nauk Matematycznych i Przyrodniczych, Prace Zakładu. Geologicznego Uniwersytetu St. Batorego w Wilnie 3: 1–49.

[mcaf143-B66] Mander L , Kürschner WM, McElwain JC. 2010. An explanation for conflicting records of Triassic-Jurassic plant diversity. Proceedings of the National Academy of Sciences of the United States of America 107: 15351–15356. 20713737 10.1073/pnas.1004207107 PMC2932585

[mcaf143-B67] Mander L , Kürschner WM, McElwain JC. 2013. Palynostratigraphy and vegetation history of the Triassic–Jurassic transition in East Greenland. Journal of the Geological Society 170: 37–46.

[mcaf143-B68] Marmi J , Tosal A, Martín-Closas C. 2023. Evolutionary history, biogeography, and extinction of the cretaceous cheirolepidiaceous conifer, *Frenelopsis*. Evolving Earth 1: 100017.

[mcaf143-B69] Marynowski L , Simoneit B. 2009. Widespread Upper Triassic to Lower Jurassic wildfire records from Poland: evidence from charcoal and pyrolytic polycyclic aromatic hydrocarbons. PALAIOS 24: 785–798.

[mcaf143-B70] Marzoli A , Renne PR, Piccirillo EM, Ernesto M, Bellieni G, Min AD. 1999. Extensive 200-million-year-old continental flood basalts of the central Atlantic Magmatic Province. Science 284: 616–618. 10213679 10.1126/science.284.5414.616

[mcaf143-B71] McArthur AD , Jolley DW, Hartley AJ, Archer SG, Lawrence HM. 2016. Palaeoecology of syn-rift topography: a late Jurassic footwall island on the Josephine Ridge, Central Graben, North Sea. Palaeogeography, Palaeoclimatology, Palaeoecology 459: 63–75.

[mcaf143-B72] McElwain JC . 2018. Paleobotany and global change: important lessons for species to biomes from vegetation responses to past global change. Annual Review of Plant Biology 69: 761–787. 10.1146/annurev-arplant-042817-040405 29719166

[mcaf143-B73] McElwain JC , Beerling DJ, Woodward FI. 1999. Fossil plants and global warming at the Triassic-Jurassic boundary. Science 285: 1386–1390. 10464094 10.1126/science.285.5432.1386

[mcaf143-B74] McElwain JC , Popa ME, Hesselbo SP, Haworth M, Surlyk F. 2007. Macroecological responses of terrestrial vegetation to climatic and atmospheric change across the Triassic/Jurassic boundary in East Greenland. Paleobiology 33: 547–573.

[mcaf143-B75] McElwain JC , Punyasena SW. 2007. Mass extinction events and the plant fossil record. Trends in Ecology & Evolution 22: 548–557. 17919771 10.1016/j.tree.2007.09.003

[mcaf143-B76] Mehlqvist K , Vajda V, Larsson LM. 2009. A Jurassic (Pliensbachian) flora from Bornholm, Denmark – a study of a historic plant-fossil collection at Lund University, Sweden. Geologiska Föreningen i Stockholm Förhandlingar 131: 137–146.

[mcaf143-B77] Mendes MM , Kvaček J. 2022. *Frenelopsis antunesii* sp. nov., a new cheirolepidiaceous conifer from the lower Cretaceous of Figueira da Foz formation in western Portugal. Review of Palaeobotany and Palynology 300: 104643.

[mcaf143-B78] Mendes MM , Kvaček J, Doyle JA. 2023. *Pseudofrenelopsis dinisii*, a new species of the extinct conifer family Cheirolepidiaceae from the probable lower Hauterivian (Cretaceous) of western Portugal. Review of Palaeobotany and Palynology 315: 104905.

[mcaf143-B79] Michelsen O . 1979. Report on the Jurassic of the Hobro No. I and Voldum No. 1 borings, Denmark. Danmarks Geologiske Undersøgelse, Årbog 1978 86: 141–149.

[mcaf143-B80] Monje-Dussán C , Martínez C, Escapa I, Madriñán S. 2016. Nuevos registros de helechos y coníferas del Cretácico Inferior de la Cuenca del Valle Superior del Magdalena, Colombia. Boletín de Geología 38: 29–42.

[mcaf143-B81] Na Y , Sun C, Wang H, et al 2021. Application of neutron tomography in studying new material of *Ixostrobus raciborski* from the middle Jurassic of Inner Mongolia, China. Geological Journal 56: 4618–4626.

[mcaf143-B82] Nathorst AG . 1878. Om floran i Skånes kolförande bildningar I. Floran vid Bjuf, första häftet. Sveriges Geologiska Undersökning. Serie C 27: 1–52.

[mcaf143-B83] Nathorst AG . 1886. Om floran i Skånes kolförande bildningar. I. Floran vid Bjuf. Sveriges Geologiska Undersokning Serie C 85: 83–116.

[mcaf143-B84] Nathorst AG . 1906. Om några ginkgoväxter från kolgrufvorna vid Stabbarp i Skåne. Kungliga Fysiografiska Sällskapets Handlingar 17: 1–15.

[mcaf143-B85] Olsen PE , Kent DV, Sues H-D, et al 2002. Ascent of dinosaurs linked to an iridium anomaly at the Triassic-Jurassic boundary. Science 296: 1305–1307. 12016313 10.1126/science.1065522

[mcaf143-B86] Olsen P , Sha J, Fang Y, et al 2022. Arctic ice and the ecological rise of the dinosaurs. Science Advances 8: eabo6342. 35776799 10.1126/sciadv.abo6342 PMC10883366

[mcaf143-B87] Pacyna G . 2013. Critical review of research on the lower Jurassic flora of Poland. Acta Palaeobotanica 53: 141–163.

[mcaf143-B88] Pacyna G , Ziaja J, Barbacka M, Pieńkowski G, Jarzynka A, Niedźwiedzki G. 2022. Early Jurassic dinosaur-dominated track assemblages, floristic and environmental changes in the holy cross mountains region, Poland. Geological Quarterly 66: 1–43.

[mcaf143-B89] Pedersen K , Lund JJ. 1980. Palynology of the plant-bearing Rhaetian to Hettangian Kap Stewart formation, Scoresby Sund, East Greenland. Review of Palaeobotany and Palynology 31: 1–69.

[mcaf143-B90] Peterffy O , Calner M, Vajda V. 2016. Early Jurassic microbial mats—a potential response to reduced biotic activity in the aftermath of the end-Triassic mass extinction event. Palaeogeography, Palaeoclimatology, Palaeoecology 464: 76–85.

[mcaf143-B91] Petersen HI , Lindström S. 2012. Synchronous wildfire activity rise and mire deforestation at the Triassic–Jurassic boundary. PLoS One 7: e47236. 23077574 10.1371/journal.pone.0047236 PMC3471965

[mcaf143-B92] Petersen HI , Lindström S, Therkelsen J, Pedersen GK. 2013. Deposition, floral composition and sequence stratigraphy of uppermost Triassic (Rhaetian) coastal coals, southern Sweden. International Journal of Coal Geology 116–117: 117–134.

[mcaf143-B93] Pieńkowski G . 1991. Liassic sedimentation in Scania, Southern Sweden: Hettangian–Sinemurian of the Helsingborg area. Facies 24: 39–85.

[mcaf143-B94] Pole M , McLoughlin S. 2017. The first Cenozoic *Equisetum* from New Zealand. Geobios 50: 259–265.

[mcaf143-B95] Pott C , McLoughlin S. 2009. Bennettitalean foliage in the Rhaetian-Bajocian (latest Triassic-Middle Jurassic) floras of Scania, southern Sweden. Review of Palaeobotany and Palynology 158: 117–166.

[mcaf143-B96] Pott C , McLoughlin S. 2011. The Rhaetian flora of Rögla, northern Scania, Sweden. Palaeontology 54: 1025–1051.

[mcaf143-B97] Rozefelds AC , Dettmann ME, Milroy AK, Hammond A, Clifford HT, Ekins M. 2019. The unexpected, recent history of horsetails in Australia. Australian Systematic Botany 32: 255–268.

[mcaf143-B98] Samylina VA , Kiritchkova AI. 1993. The genus *Czekanowskia* Heer: principles of systematics, range in space and time. Review of Palaeobotany and Palynology 79: 271–284.

[mcaf143-B99] Schoepfer SD , Algeo TJ, van de Schootbrugge B, Whiteside JH. 2022. The Triassic-Jurassic transition – a review of environmental change at the dawn of modern life. Earth-Science Reviews 232: 104099.

[mcaf143-B100] Schweitzer H-J . 1977. Die Rhato-Jurassischen Floren des Iran und Afganistans 4. Die ratische Zwitterblute *Irania hermaphroditica* nov. spec. und ihre Bedenirtrung fur die Phylogenie du Angiospermen. Palaeontographica Abteilung B 161: 98–145.

[mcaf143-B101] Sepkoski JJ . 1981. A factor analytic description of the Phanerozoic marine fossil record. Paleobiology 7: 36–53.

[mcaf143-B102] Seward AC . 1910. Fossil plants, Vol. 2. Cambridge: Cambridge University Press.

[mcaf143-B103] Seward AC . 1919. Ginkgoales, Coniferales, Gnetales. Fossil plants: a textbook for students of Botany and Geology. Vol. 4. Cambridge: Cambridge University Press.

[mcaf143-B104] Shi G , Herrera F, Herendeen PS, et al 2017. Leaves of *Podozamites* and *Pseudotorellia* from the early Cretaceous of Mongolia: stomatal patterns and implications for relationships. Journal of Systematic Palaeontology 16: 111–137.

[mcaf143-B105] Sivhed U . 1984. Litho- and biostratigraphy of the upper Triassic–middle Jurassic in Scania, southern Sweden. Sveriges Geologiska Undersökning C, No. 806.

[mcaf143-B106] Slater SM , McKie T, Vieira M, Wellman CH, Vajda V. 2017. Episodic river flooding events revealed by palynological assemblages in Jurassic deposits of the Brent group, North Sea. Palaeogeography, Palaeoclimatology, Palaeoecology 485: 389–400.

[mcaf143-B107] Slodownik M , Vajda V, Steinthorsdottir M. 2021. Fossil seed fern *Lepidopteris ottonis* from Sweden records increasing CO2 concentration during the end-Triassic extinction event. Palaeogeography, Palaeoclimatology, Palaeoecology 564: 110157.

[mcaf143-B108] Sun C , Dilcher DL, Wang H, Sun G, Ge Y. 2009. *Czekanowskia* from the Jurassic of Inner Mongolia, China. International Journal of Plant Sciences 170: 1183–1194.

[mcaf143-B109] Sun C , Li Y, Dilcher DL, et al 2015. An introductory report on the biodiversity of middle Jurassic *Phoenicopsis* (Czekanowskiales) from the Ordos Basin, China. Science Bulletin 60: 1858–1865.

[mcaf143-B110] Sun G , Zheng S, Dilcher DL, Wang Y, Mei S. 2001. Early angiosperms and their associated plants from Western Liaoning, China. Shanghai: Shanghai Scientific and Technological Education Publishing House.

[mcaf143-B111] Taylor TN , Taylor EL, Krings M. 2009. Paleobotany: the biology and evolution of fossil plants. Englewood Cliffs: Prentice Hall.

[mcaf143-B112] Tosolini A-MP , McLoughlin S, Wagstaff BE, Cantrill DJ, Gallagher SJ. 2015. Cheirolepidiacean foliage and pollen from Cretaceous high-latitudes of southeastern Australia. Gondwana Research 27: 960–977.

[mcaf143-B113] Tralau H . 1966. Botanical investigations into the fossil flora of Eriksdal in Fyledalen, Scania. Sveriges Geologiska Undersökning Ser C, No. 611.

[mcaf143-B114] Troedsson G . 1947. Berggrunden inom Hälsingborgs stad. Geologiska Föreningen i Stockholm Förhandlingar 69: 385–432.

[mcaf143-B115] Troedsson G . 1951. On the Höganäs Series of Sweden (Rhaeto-Lias). Kungliga Fysiografiska Sällskapets Handlingar 62: 1–269.

[mcaf143-B116] Turutanova-Ketova AI . 1963. Semejstvo Cheirolepidiaceae (=Cheirolepidaceae) Hirmer und Hörhammer, 1934. In: Osnovy paleontologii. Vol. 15. Moscow: Izdatel’stvo Akademii Nauk SSSR.

[mcaf143-B117] Unverfärth J , McLoughlin S, Möllmann M, Bomfleur B. 2022. *Sphenobaiera insecta* from the upper Triassic of South Australia, with a clarification of the genus *Sphenobaiera* (fossil Ginkgophyta) and its delimitation from similar foliage genera. Botany Letters 169: 442–453.

[mcaf143-B118] Vajda V . 2001. Aalenian to Cenomanian terrestrial palynofloras of SW Scania, Sweden. Acta Palaeontologica Polonica 46: 403–426.

[mcaf143-B119] Vajda V , Bercovici A. 2014. The global vegetation pattern across the Cretaceous–Paleogene mass extinction interval: a template for other extinction events. Global and Planetary Change 122: 29–49.

[mcaf143-B120] Vajda V , Calner M, Ahlberg A. 2013. Palynostratigraphy of dinosaur footprint-bearing deposits from the Triassic–Jurassic boundary interval of Sweden. Geologiska Föreningen i Stockholm Förhandlingar 135: 120–130.

[mcaf143-B121] Vajda V , Kear BP. 2024. An earliest Triassic riparian ecosystem from the Bulgo Sandstone (Sydney Basin), Australia: palynofloral evidence of a high-latitude terrestrial vertebrate habitat after the end-Permian mass extinction. Alcheringa 48: 483–494.

[mcaf143-B122] Vajda V , Linderson H, Mcloughlin S. 2016. Disrupted vegetation as a response to Jurassic volcanism in southern Sweden. Journal of the Geological Society 434: 127–147.

[mcaf143-B123] Vajda V , McLoughlin S, Slater SM, Gustafsson O, Rasmusson AG. 2023. The ‘seed-fern’ *Lepidopteris* mass-produced the abnormal pollen *Ricciisporites* during the end-Triassic biotic crisis. Palaeogeography, Palaeoclimatology, Palaeoecology 627: 111723. Licensed under Creative Commons CC BY 4.0 Attribution International licence. https://creativecommons.org/licenses/by/4.0/.

[mcaf143-B124] Vajda V , Wigforss-Lange J. 2009. Onshore Jurassic of Scandinavia and related areas. Geologiska Föreningen i Stockholm Förhandlingar 131: 5–23.

[mcaf143-B125] Vakhrameev VA . 1987. Climates and the distribution of some gymsosperms in Asia during the Jurassic and Cretaceous. Review of Palaeobotany and Palynology 51: 205–212.

[mcaf143-B126] van de Schootbrugge B , Quan TM, Lindström S, et al 2009. Floral changes across the Triassic/Jurassic boundary linked to flood basalt volcanism. Nature Geoscience 2: 589–594.

[mcaf143-B127] van de Schootbrugge B , Wignall PB. 2016. A tale of two extinctions: converging end-Permian and end-Triassic scenarios. Geological Magazine 153: 332–354.

[mcaf143-B128] Van Konijnenburg-van Cittert JHA . 1971. In situ gymnosperm pollen from the Middle Jurassic of Yorkshire. Acta Botanica Neerlandica 20: 1–97.

[mcaf143-B129] Van Konijnenburg-van Cittert JHA , Pott C, Schmeißner S, Dütsch G, Kustatscher E. 2021. The Rhaetian flora of Wüstenwelsberg, Bavaria, Germany: description of selected gymnosperms (Ginkgoales, Cycadales, Coniferales) together with an ecological assessment of the locally prevailing vegetation. Review of Palaeobotany and Palynology 288: 104398.

[mcaf143-B130] van Konijnenburg-van Cittert JHA , Schmeißner S, Dütsch G, Kustatscher E, Pott C. 2022. Plant macrofossils from the Rhaetian of Einberg near Coburg (Bavaria, Germany). Part 2. Cycadophyta and Ginkgophyta. Neues Jahrbuch für Geologie und Paläontologie-Abhandlungen 305: 109–130.

[mcaf143-B131] Wang Y , Guignard G, Thévenard F, et al 2005a. Cuticular anatomy of *Sphenobaiera huangii* (Ginkgoales) from the lower Jurassic of Hubei, China. American Journal of Botany 92: 709–721. 21652450 10.3732/ajb.92.4.709

[mcaf143-B132] Wang Y , Mosbrugger V, Zhang H. 2005b. Early to middle Jurassic vegetation and climatic events in the Qaidam Basin, Northwest China. Palaeogeography, Palaeoclimatology, Palaeoecology 224: 200–216.

[mcaf143-B133] Watson J . 1988. The Cheirolepidiaceae. In: Beck CB. ed. Origin and evolution of gymnosperms. New York: Columbia University Press, 382–447.

[mcaf143-B134] Weber R . 1968. Die fossile flora der Rhaet-Lias-Ubergangsschichten von Bayreuth (Oberfranken) unter besonderer Beruecksichtigung der Coenologie. Erlanger Geologische Abhandlungen 72: 1–73.

[mcaf143-B135] Weibel R , Lindström S, Pedersen GK, et al 2016. Groundwater table fluctuations recorded in zonation of microbial siderites from end-Triassic strata. Sedimentary Geology 342: 47–65.

[mcaf143-B136] Wesley A . 1965. The fossil flora of the grey limestones of Veneto northern Italy, and its relationships to the other European floras of similar age. Journal of Palaeosciences 14: 124–130.

[mcaf143-B137] Wesley A . 1974. On the bennettitalean remains from the Lias of northern Italy. In: *Symposium on morphological and stratigraphical palaeobotany. Birbal Sahni Institute of Palaeobotany, Lucknow, Special Publication* 2: 66–71.

[mcaf143-B138] Whiteside JH , Olsen PE, Kent DV, Fowell SJ, Et-Touhami M. 2007. Synchrony between the Central Atlantic magmatic province and the Triassic–Jurassic mass-extinction event? Palaeogeography, Palaeoclimatology, Palaeoecology 244: 345–367.

[mcaf143-B139] Wignall PB , Atkinson JW. 2020. A two-phase end-Triassic mass extinction. Earth-Science Reviews 208: 103282.

[mcaf143-B140] Yang X-J , Friis EM, Zhou Z-Y. 2008. Ovule-bearing organs of *Ginkgo ginkgoidea* (Tralau) comb. nov., and associated leaves from the Middle Jurassic of Scania, South Sweden. Review of Palaeobotany and Palynology 149: 1–17.

[mcaf143-B141] Zavialova N , Nosova N, Bugdaeva E. 2023. On the exine ultrastructure of fossil ginkgoaleans: *in situ* pollen of *Sorosaccus* Harris. Review of Palaeobotany and Palynology 313: 104838.

[mcaf143-B142] Zhou Z-Y . 2009. An overview of fossil Ginkgoales. Palaeoworld 18: 1–22.

