# Supplementary material for: Earliest Jurassic plant assemblages from Sweden reveal a low-diversity ginkgoalean and cheirolepid flora dominating the post-extinction landscape
Source: Ann Bot. 2025 Jul 9;137(6):1797–816. doi: 10.1093/aob/mcaf143 (PMC13275009; doi:10.1093/aob/mcaf143)
Supplement: mcaf143_Supplementary_Data [file mcaf143_supplementary_data.docx]

**SUPLEMMENTARY INFORMATION**

Table S1. Comparison of Boserup beds flora with Earliest Hettangian, Hettangian–Sinemurian assemblages from East Greenland, Poland, Hungary and Germany.

| Main groups | Taxa | Scoresby Sound, East Greenland (Harris, 1937) | | Holy Cross Mountains, Poland (Pacyna, 2013, 2022; Barbacka *et al*. 2022) | | Boserup beds, Skåne, Sweden (this study) | Mecsek Mountains, southern Hungary (Barbacka, 2011) | Bavaria, Germany (Van Konijnenburg-van Cittert *et al.* 2021) |
| --- | --- | --- | --- | --- | --- | --- | --- | --- |
|  |  | (*Thaumatopteris* zone, first 25 meters) | Zagaje Fm. (Lower Hettangian) | | Höganäs Fm. (Lower Hettangian) | | Mecsek Coal Fm. (Hettangian–Sinemurian) | (Hettangian) |
| Lycophytes | *Equisetites laevis* | X |  | |  | |  |  |
|  | *Equisetites sarrani* | X |  | |  | |  |  |
|  | *Equisetites columnaris* |  |  | |  | | X |  |
|  | *Equisetites muensteri* |  | X | |  | | X | X |
|  | *Equisetites* sp*.* |  | X | |  | | X |  |
|  | *Lycostrobus scottii* | X |  | |  | |  |  |
|  | *Odrolepis liassica* |  | X | |  | |  |  |
| Sphenophytes | *Neocalamites* sp. |  |  | | X | |  |  |
|  | *Neocalamites carcinoides* |  |  | |  | | X |  |
|  | *Schizoneura carcinoides* |  |  | |  | |  | X |
|  | *Schizoneura* sp. |  | X | |  | |  |  |
|  | *Neocalamites lehmannianus* |  | X | |  | |  | X |
| Ferns | *Cladophlebis* spp. |  |  | | X | |  |  |
|  | *Cladophlebis denticulata* |  |  | |  | | X |  |
|  | *Cladophlebis haiburnensis* |  |  | |  | | X |  |
|  | *Cladophlebis roesserti* |  |  | |  | | X |  |
|  | *Clathropteris meniscoides* | X |  | |  | | X | X |
|  | *Coniopteris hymenophylloides* |  | X | |  | | X |  |
|  | *Dictyophyllum* aff. *dunkeri* |  | X | |  | |  |  |
|  | *Dictyophyllum* an nova spec.? |  | X | |  | |  |  |
|  | *Dictyophyllum muensteri* |  |  | |  | |  | X |
|  | *Dictyophyllum nilssoni* | X | X | |  | | X | X |
|  | *Dictyophyllum rugosum* |  |  | |  | | X |  |
|  | *Dictyophyllum* sp. |  | X | |  | |  |  |
|  | *Goeppertella microloba* |  | X | |  | |  | X |
|  | *Laccopteris elegans* |  | X | |  | |  |  |
|  | *Marattia intermedia* |  |  | |  | |  | X |
|  | *Marattiopsis muensteri* |  | X | |  | |  |  |
|  | *Marattiopsis hoerensis* |  |  | |  | | X |  |
|  | *Pecopteris concinna* |  | X | |  | |  |  |
|  | *Phialopteris heterophylla* |  |  | |  | |  | X |
|  | *Phlebopteris angustiloba* |  | X | |  | | X | X |
|  | *Phlebopteris muensteri* | X |  | |  | |  | X |
|  | *Phlebopteris* sp. |  |  | |  | | X |  |
|  | *Rhaphidopteris duetschii* |  |  | |  | |  | X |
|  | *Rhizomopteris* |  | X | |  | |  |  |
|  | *Selenocarpus muensterianus* |  |  | |  | |  | X |
|  | *Sphenopteris* sp. |  |  | |  | | X |  |
|  | *Todites goeppertianus* | X |  | |  | | X |  |
|  | *Todites princeps* | X | X | |  | | X | X |
|  | *Todites princeps forma trilobata* |  | X | |  | |  |  |
|  | *Todites roessertii* |  |  | |  | |  | X |
|  | *Todites williamsoni* |  | X | |  | |  |  |
|  | *Thaumatopteris brauniana* | X | X | |  | | X | X |
|  | *Thaumatopteris schenkii* | X | X | |  | |  |  |
| Caytoniales | *Caytonia* sp. |  | X | |  | |  |  |
|  | *Komlopteris nordenskioeldii* |  |  | |  | | X |  |
|  | *Pachypteris rhomboidalis* |  |  | |  | |  | X |
|  | *Pachypteris saligna* |  |  | |  | |  | X |
|  | *Pachypteris banatica* |  |  | |  | | X |  |
|  | *Pachypteris lanceolata* |  | X | |  | |  |  |
|  | *Sagenopteris hallei* |  |  | |  | | X |  |
|  | *Sagenopteris nilssoniana* | X | X | |  | | X | X |
|  | *Sagenopteris pilosa* |  |  | |  | | X |  |
|  | *Sagenopteris rhoifolia* var. *pusilla* |  | X | |  | |  |  |
| Cycadales | *Bjuvia simplex* |  |  | |  | | X |  |
|  | *Ctenis* sp. |  |  | |  | | X |  |
|  | *Cycadites*sp. |  |  | |  | |  | X |
|  | *Nilssonia acuminata* |  |  | |  | |  | X |
|  | *Nilssonia minima* |  |  | |  | |  | X |
|  | *Nilssonia orientalis* |  | X | |  | |  |  |
|  | *Nilssonia obtusa* |  |  | |  | | X |  |
|  | *Nilssonia polymorpha* |  |  | |  | | X | X |
|  | *Nilssonia* sp. |  | X | |  | |  |  |
|  | *Nilssonia revoluta* |  |  | |  | | X |  |
|  | *Nilssoniopteris*sp. |  |  | |  | |  | X |
|  | *Otozamites brevifolius* |  | X | |  | |  | X |
|  | *Paracycas minuta* |  | X | |  | |  |  |
|  | *Pseudoctenis spectabilis* | X |  | |  | |  |  |
|  | *Pseudoctenis prossii* |  |  | |  | |  | X |
|  | *Pseudoctenis* sp. |  |  | |  | | X |  |
|  | *Taeniopteris superba* |  | X | |  | |  |  |
|  | *Taeniopteris tenuinervis* |  | X | |  | |  |  |
|  | *Zamites*sp. |  |  | |  | |  | X |
| Bennettitales | *Anomozamites hartzi* |  |  | |  | |  |  |
|  | *Anomozamites gracilis* |  |  | |  | |  | X |
|  | *Anomozamites marginatus* | X |  | |  | | X | X |
|  | *Pterophyllum alinae* |  | X | |  | |  |  |
|  | *Pterophyllum* sp. |  | X | |  | |  | X |
|  | *Pterophyllum subaequale* | X |  | |  | | X |  |
|  | *Weltrichia mirabilis* |  |  | |  | |  | X |
| Ginkgoales | *Baiera* sp. |  | X | |  | |  |  |
|  | *Baiera furcata* |  |  | |  | | X |  |
|  | *Geinitzia* sp. |  |  | |  | | X |  |
|  | *Ginkgoites hermelini* | X |  | |  | |  |  |
|  | *Ginkgoites taeniatus* |  |  | |  | |  | X |
|  | *Ginkgoites sp. cf. G. marginatus* |  |  | | X | | X |  |
|  | *Ginkgoites minuta* |  |  | |  | | X |  |
|  | *Ginkgo* aff. *whittbyensis* |  | X | |  | |  |  |
|  | *Karkenia hauptmannii* |  |  | |  | |  | X |
|  | *Schmeissneria microstachys* |  | X | |  | |  | X |
|  | *Sphenobaiera paucipartita* |  |  | | X | |  |  |
|  | *Sphenobaiera spectabilis* | X |  | |  | |  | X |
|  | *Sphenobaiera leptophylla* |  |  | |  | | X |  |
|  | *Sphenobaiera longifolia* |  |  | |  | | X |  |
|  | *Sorosaccus gracilis* |  |  | | X | |  |  |
|  | *Stachyopitys preslii* |  |  | |  | |  | X |
| Czekanowskiales | *Czekanowskia* sp.  *Czekanowskia nathorstii*  *Czekanowskia rigida*  *Ixostrobus* sp.  *Ixostrobus siemiradzkii* | X | X  X  X | | X | |  |  |
| Conifers | *Brachyphyllum* sp. cf. *B. crucis*  *Brachyphyllum* sp. |  | X | | X | |  |  |
|  | *Brachyphyllum crucis* |  |  | |  | | X |  |
|  | *Brachyphyllum mamillare* |  |  | |  | | X |  |
|  | *Brachyphyllum papareli* |  |  | |  | | X |  |
|  | *Elatocladus* sp. |  |  | |  | | X |  |
|  | *Hirmeriella airelensis* |  |  | |  | | X |  |
|  | *Hirmeriella muensteri* |  | X | |  | |  | X |
|  | *Palissya sphenolepis* |  |  | |  | |  | X |
|  | *Pagiophyllum ordinatum* |  |  | |  | | X |  |
|  | *Pagiophyllum peregrinum* |  |  | |  | | X |  |
|  | *Podozamites distans* |  |  | |  | |  | X |
|  | *Podozamites lanceolatus* |  |  | |  | | X |  |
|  | *Podozamites* cf. *schenkii* |  | X | |  | |  | X |
|  | *Podozamites* sp. |  | X | |  | | X |  |
|  | *Pseudotorellia sp.* |  |  | | X | |  |  |
|  | *Schizolepis follinii* |  | X | |  | |  |  |
|  | *Schizolepis liasokeuperianus* |  |  | |  | |  | X |
|  | *Stachyotaxus septentrionalis* |  | X | |  | |  |  |
|  | *Swedenborgia* sp. | X | X | |  | |  | X |
| Gnetales | *Bernettia inopinata* |  |  | |  | |  | X |
|  | *Piroconites kuespertii* |  | X | |  | |  | X |
| Conifers *incertae sedis* | *Bysmatospermum macrotrachelum*  *Desmiophyllum gothanii*  *Desmiophyllum* sp.  *Campylophyllum hoermannii*  *Chlamydolepis lautneri* | X | X | |  | | X | X  X  X |
| Seed-fern | *Ctenozamites (Ptilozamites) wolffiana* |  |  | |  | |  | X |
|  | *Komlopteris* sp. |  | X | |  | |  |  |
|  | *Pachypteris lanceolata* |  | X | |  | |  |  |
|  | *Pachypteris papilosa* |  | X | |  | |  |  |
|  | *Ptilozamites cycadea* |  | X | |  | | X |  |
|  | *Stenopteris dinosaurensis* | X |  | |  | |  |  |
| *Incertae sedis* | *Allicospermum* sp. |  |  | | X | |  |  |
|  | *Acrostichites hartzi* | X |  | |  | |  |  |
|  | *Amphorispermum major* | X |  | |  | |  |  |
|  | *Ourostrobus nathorsti* | X |  | |  | |  |  |
